# Hypoxia-induced metabolic and apoptotic reprogramming enhances immunomodulation in Wharton’s jelly mesenchymal stem cells

**DOI:** 10.1016/j.isci.2026.115363

**Published:** 2026-03-13

**Authors:** Mohini Mendiratta, Meenakshi Mendiratta, Sujata Mohanty, Hridayesh Prakash, Lakshay Malhotra, Sandeep Rai, Vijaya Sarangathem, Sabyasachi Bandyopadhyay, GuruRao Hariprasad, Ritu Gupta, Sameer Bakhshi, Vatsla Dadhwal, Deepam Pushpam, Mukul Aggarwal, Aditya Kumar Gupta, Prabhat Singh Malik, Raja Pramanik, Manoranjan Mahapatra, Tulika Seth, Rishi Dhawan, Baibaswata Nayak, Thoudam Debraj Singh, Sachin Kumar, Riyaz Ahmed Mir, Surender Kumar Sharawat, Ranjit Kumar Sahoo

**Affiliations:** 1 Department of Medical Oncology, Dr. B. R. Ambedkar Institute Rotary Cancer Hospital, All India Institute of Medical Sciences, New Delhi 110029, India; 2 Stem Cell Facility (DBT-Centre of Excellence for Stem Cell Research), All India Institute of Medical Sciences, New Delhi 110029, India; 3 Amity Centre for Translational Research, Amity University, Sector 125, Noida 201313, India; 4 Department of Biochemistry, Sri Venkateswara College, University of Delhi, New Delhi 110021, India; 5 Laboratory Oncology Unit, Dr. B. R. Ambedkar Institute Rotary Cancer Hospital, All India Institute of Medical Sciences, New Delhi 110029, India; 6 Department of Biochemistry and Cell Biology, Cell & Matrix Research Institute, Kyungpook National University, School of Medicine, Daegu 41944, Republic of Korea; 7 Proteomics Sub-facility, Centralized Core Research Facility, All India Institute of Medical Sciences, New Delhi 110029, India; 8 Department of Biophysics, All India Institute of Medical Sciences, New Delhi 110029, India; 9 Department of Obstetrics and Gynecology, All India Institute of Medical Sciences, New Delhi 110029, India; 10 Department of Hematology, All India Institute of Medical Sciences, New Delhi 110029, India; 11 Department of Pediatric Oncology, All India Institute of Medical Sciences, New Delhi 110029, India; 12 Department of Gastroenterology and Human Nutrition, All India Institute of Medical Sciences, New Delhi 110029, India; 13 Department of Biochemistry, All India Institute of Medical Sciences, New Delhi 110029, India

**Keywords:** immunology, cell biology, stem cells research

## Abstract

Mesenchymal stem cells (MSCs) possess immunomodulatory properties that can be harnessed for treating acute graft-versus-host disease (aGVHD). In this study, we compared bone marrow (BM) and Wharton’s jelly (WJ)-derived MSCs and investigated how hypoxia preconditioning (1% O_2_, 24 h) influences their immunoregulatory function. Using direct co-cultures with activated peripheral blood mononuclear cells from aGVHD patients, we evaluated T cell proliferation, Treg induction, macrophage polarization, mitochondrial transfer, and MSC apoptosis. Hypoxia-preconditioned WJ-MSCs (WJ-MSCs^HYP^) more effectively suppressed T cell proliferation, enhanced Treg differentiation, promoted M2 macrophage polarization, and improved T cell metabolic balance via mitochondrial transfer. These effects were primarily driven by apoptosis and occurred independently of efferocytosis. Our findings highlight tissue-specific mechanisms underlying MSCs’ immunoregulation and reveal that hypoxia enhances the therapeutic potential of WJ-MSCs. This work provides mechanistic insight into MSCs-based interventions and supports WJ-MSCs^HYP^ as a promising cell source for immunomodulatory therapy in inflammatory disorders such as aGVHD.

## Highlights

• Hypoxia preconditioning enhances the immunomodulatory function of MSCs
• WJ-MSCs^HYP^ outperform BM-MSCs^HYP^ in T cell suppression, Treg, and M2 induction
• Mitochondrial transfer by WJ-MSCs^HYP^ reduces ROS and improves T cell metabolism
• Hypoxia-induced apoptosis enhances immunomodulation of MSCs independent of efferocytosis

## Graphical abstract

**Figure:**
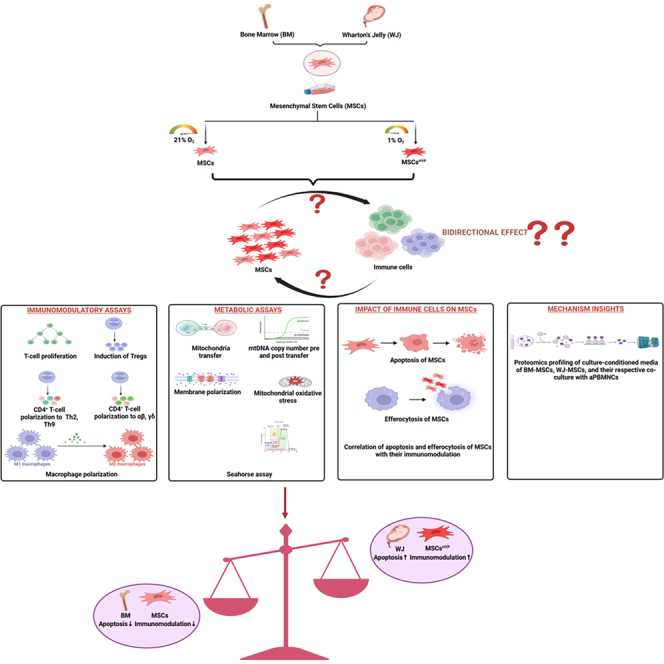

## Introduction

Allogeneic hematopoietic stem cell transplantation (Allo-HSCT) offers curative potential for hematological disorders, but its success is often offset by acute graft-versus-host disease (aGVHD), a life-threatening complication driven by donor T cell-mediated immune attack on host tissues, particularly the skin, liver, and gastrointestinal system.^1^ aGVHD occurs in up to 40%–60% of recipients and involves extreme activation of inflammatory mechanisms by T helper type 1/T helper type 17 (Th1/Th17) cytokine responses and cytotoxic effector mechanisms.^2^

Mesenchymal stem cells (MSCs) are promising immunoregulatory agents for aGVHD, with preclinical studies demonstrating their capacity to suppress T cell activation, inhibit dendritic cell maturation, modulate natural killer (NK)- and B cell activity, and release diverse immunomodulatory mediators, such as cytokines, chemokines, and extracellular vesicles inhibitory, NK- and B cell-modulating, and secretion of diverse immunomodulatory molecules, such as chemokines, cytokines, and extracellular vesicles/exosomes^3^^,^^4^; however, outcomes from large-scale clinical trials remain inconsistent,^5^ underscoring gaps in understanding their mechanisms of action.

Increasing evidence indicates that the therapeutic efficacy of MSCs is not based on their persistence *in vivo*, but on their capacity to undergo apoptosis after identification by immune effector cells such as cytotoxic T cell, NK cells, and granulocytes. Notably, experiments silencing central apoptotic molecules such as Bcl-2-associated X protein (BAX) and Bcl-2 Antagonist/Killer 1 (BAK) affirmed that avoiding MSCs’ apoptosis reduces their immunomodulatory effect, determining apoptotic turnover as a requirement for therapeutic effectiveness.^6^ The apoptotic MSCs are then taken up by host macrophages by efferocytosis, and this process licenses macrophages to release immunoregulatory mediators.^6^^,^^7^^,^^8^

Whereas the majority of studies utilized bone marrow (BM)-derived MSCs (BM-MSCs), recent data show that Wharton’s jelly (WJ)-derived MSCs (WJ-MSCs) are superior to BM-MSCs in terms of immunomodulatory and survival ability, partly because of their primitive developmental origin and greater ability to secrete trophic factors.^6^^,^^9^^,^^10^ The scalability of sourcing of WJ-MSCs also places them as a highly viable candidate for clinical use in aGVHD. However, direct comparisons of how their immune regulatory mechanisms differ under Allo-HSCT-related stressors, such as the hypoxic conditions of aGVHD, remain limited.

Hypoxia preconditioning of MSCs, which mimics the low-oxygen conditions where MSCs naturally reside,^11^ enhances their survival, paracrine activity, and immunosuppressive function by upregulating mediators such as indoleamine 2,3 dioxygenase (IDO), transforming growth factor β (TGF-β), and prostaglandin (PGE_2_).^12^^,^^13^^,^^14^ In addition, hypoxia triggers metabolic reprogramming of MSCs, converting their bioenergetic status from oxidative phosphorylation to glycolysis. This metabolic adjustment not only increases their resistance to aggressive inflammatory environments (e.g., aGVHD) but also directly affects their immunomodulatory response. Current evidence shows that glycolytic reprogramming during hypoxia boosts the release of immunoregulatory metabolites and extracellular vesicles by MSCs, as well as modulates mitochondrial dynamics that regulate apoptosis and efferocytosis.^15^^,^^16^^,^^17^ Hence, hypoxia-induced bioenergetic remodeling can be a central determinant of how MSCs interact with host immune cells.

Collectively, apoptosis, hypoxia, and metabolic programming come into view as a master axis governing the immunobiology of MSCs. In this study, we systematically compared BM-MSCs and WJ-MSCs to assess how hypoxia preconditioning shapes their metabolism, immunoregulatory functions, and interactions with immune effectors. Using immune assays, mitochondrial bioenergetic profiling, and comparative proteomics, we aimed to define how tissue origin and hypoxic adaptation gain a mechanistic understanding of how tissue origin and hypoxic adaptation influence MSCs’ performance within aGVHD.

## Results

### Hypoxia preconditioning of MSCs preserved their parental identity

Human MSCs isolated from both BM and WJ retained their parental identity following hypoxic preconditioning (1% O_2_, 24 h). These cells maintained their characteristic spindle-shaped fibroblast-like morphology (Figure S1A) and expressed ≥95% of the surface markers CD105, CD73, CD90, CD29, and HLA-I, with minimal or no expression (≤2%) of lineage-specific markers such as CD34 and CD45, as well as HLA-II (Figure S1B). Additionally, both MSCs populations demonstrated their *in vitro* potential to differentiate into mesodermal lineages (Figure S1C). MSCs exhibited a normal karyotype, as confirmed by chromosomal analysis (Figure S1D).

### WJ-MSCs^HYP^ inhibited CD3^+^ T cell proliferation, polarized effector CD4^+^ T cell to a regulatory and anti-inflammatory phenotype

**Figure 1:**
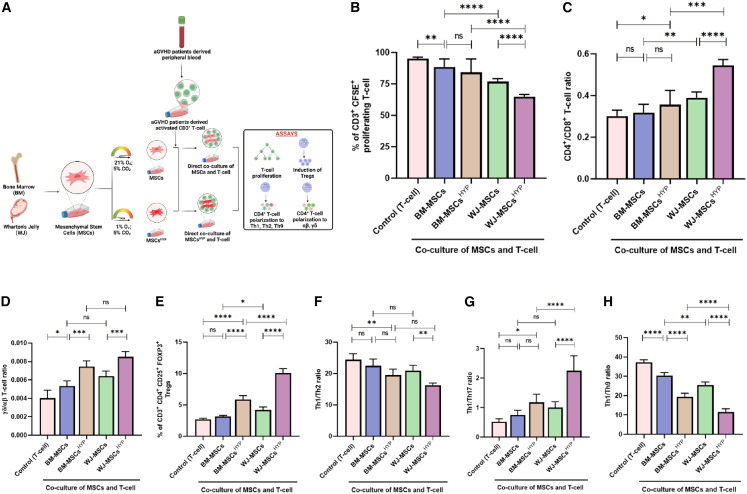
Effect of naive and hypoxia-preconditioned MSCs (MSCs, MSCs^HYP^) on the aGVHD patients-derived T cell reprogramming (A) Schematic representation of the co-culture model for the assessment of T cell programming. (B) The bar graph represents the percentage of CD3^+^ CFSE^+^ proliferating T cell (*n* = 25). (C) The ratio of CD4^+^/CD8^+^ T cell (*n* = 25). (D) The ratio of γδ/αβ CD4^+^ T cell (*n* = 25). (E) The percentage of CD3^+^ CD4^+^ CD25^+^ FOXP3^+^ Tregs (*n* = 25). (F) The ratio of Th1/Th2 (*n* = 25). (G) The ratio of Th1/Th17 (*n* = 25). (H) The ratio of Th1/Th9 (*n* = 25) in the direct co-culture of MSCs and T cell. Data shown represent the mean ± SD of 25 independent experiments performed with T cell derived from 25 different donors (biological replicates), with each experiment conducted in triplicate (technical replicates). Statistical analysis: Tukey’s multiple comparisons test; ∗≤0.05; ∗∗≤0.01; ∗∗∗≤0.001; ∗∗∗∗≤0.0001. BM, bone marrow; WJ, Wharton’s jelly; MSCs, mesenchymal stem cells; HYP, hypoxia-preconditioned.

The immunomodulatory effects of naive and hypoxia-preconditioned BM- and WJ-derived MSCs (MSCs^HYP^) were assessed in direct co-culture with aGVHD patient CD3^+^ T cell to evaluate their impact on T cell proliferation and polarization toward regulatory phenotypes (Figure 1A).

Both MSCs inhibited CD3^+^ T cell proliferation compared to control, with WJ-MSCs being more effective than BM-MSCs (*p* ≤ 0.0001). Hypoxia preconditioning further enhanced immunosuppression, with WJ-MSCs^HYP^ showing the greatest effect, significantly outperforming both WJ-MSCs and BM-MSCs^HYP^ (*p* ≤ 0.0001), while no statistically significant impact of hypoxia was observed on BM-MSCs (*p* = 0.1153) (Figure 1B; Table S1). The baseline clinical and demographic characteristics of aGVHD patients included in this study, summarized in Table S2.

There was no significant difference in the CD4^+^/CD8^+^ T cell ratio between control and BM-MSCs (*p* = 0.9138) or between BM-MSCs and BM-MSCs^HYP^ (*p* = 0.2387). In contrast, WJ-MSCs and WJ-MSCs^HYP^ significantly increased the CD4^+^/CD8^+^ ratio compared to their BM counterparts (*p* = 0.0034 and *p* ≤ 0.001, respectively) (Figure 1C; Table S1).

In direct co-culture, MSCs reduced the proportion of αβ CD4^+^ T cell and increased the γδ subset. Hypoxia-preconditioned MSCs from both BM and WJ were significantly more effective than their naive forms (*p* = 0.0003 for each). Although WJ-MSCs^HYP^ appeared superior to BM-MSCs^HYP^ in increasing γδ T cell, the difference was not statistically significant (*p* = 0.0982), indicating both sources are comparably effective after hypoxia preconditioning (Figure 1D; Table S1).

MSCs increased Tregs generation, with BM-MSCs showing no significant effect over control (*p* = 0.5756), while BM-MSCs^HYP^ significantly enhanced Tregs induction (*p* ≤ 0.0001). WJ-MSCs^HYP^ were markedly more effective than WJ-MSCs (*p* ≤ 0.0001) (Figures 1E, S4A, and S4B; Table S1). MSCs also promoted Th2 polarization, as reflected by a reduced Th1/Th2 ratio, with WJ-MSCs^HYP^ exerting a stronger effect than WJ-MSCs (*p* ≤ 0.01); no significant difference was seen between BM-MSCs and controls or BM-MSCs^HYP^ (Figure 1F; Table S1). Also, WJ-MSCs^HYP^ significantly decreased the Th1/Th17 ratio compared to BM-MSCs^HYP^ (*p* ≤ 0.0001) (Figure 1G; Table S1). Both MSCs and MSCs^HYP^ polarized CD4^+^ T cell toward a Th9 phenotype, with WJ-MSCs and WJ-MSCs^HYP^ outperforming their BM counterparts (*p* = 0.0011 and *p* ≤ 0.0001, respectively) (Figure 1H; Table S1).

### WJ-MSCs^HYP^ promoted macrophage polarization toward the M2 phenotype

**Figure 2:**
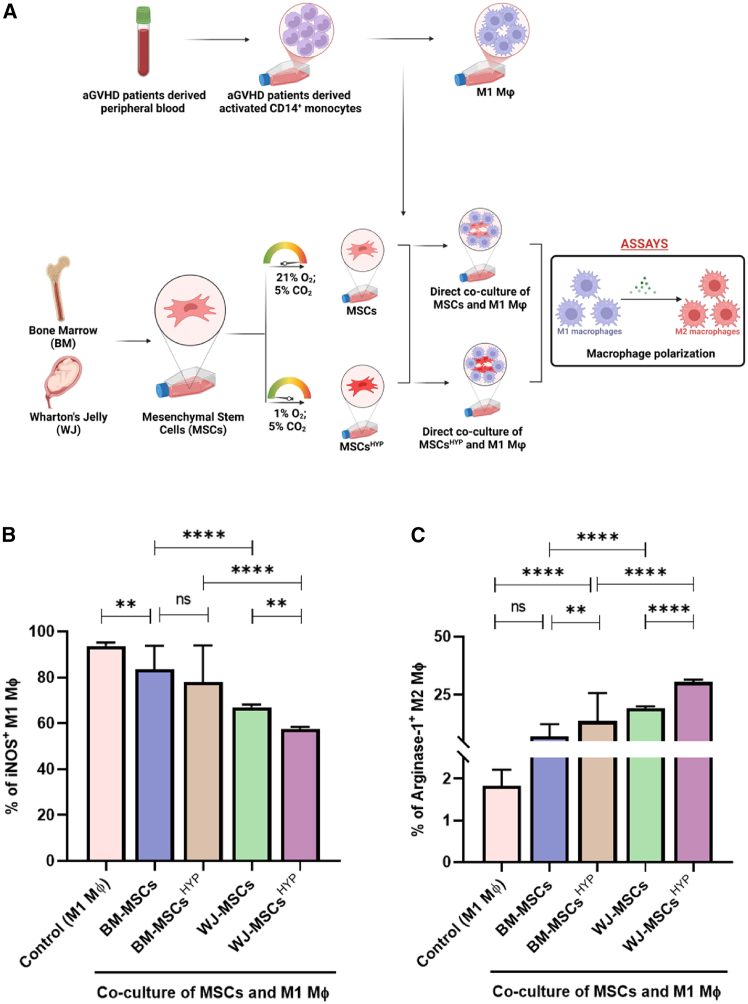
Effect of naive and hypoxia-preconditioned MSCs (MSCs, MSCs^HYP^) on the antigen-presenting cells of aGVHD patients (A) Diagrammatic representation of the co-culture model for the evaluation of impact of MSCs/MSCs^HYP^ on antigen-presenting cells. (B) The bar graph represents the percentage of iNOS^+^ M1 MФ. (C) The percentage of arginase-1^+^ M2 MФ in the direct co-culture of MSCs and M1 MФ. Data shown represent the mean ± SD of 25 independent experiments performed with mDCs/M1 MФ derived from 25 different donors (biological replicates), with each experiment conducted in triplicate (technical replicates). Statistical analysis: Tukey’s multiple comparisons test; ∗≤0.05; ∗∗≤0.01; ∗∗∗≤0.001; ∗∗∗∗≤0.0001. BM, bone marrow; WJ, Wharton’s jelly; MSCs, mesenchymal stem cells; HYP, hypoxia-preconditioned; mDCs, mature dendritic cells.

Both MSCs and hypoxia-preconditioned MSCs (MSCs^HYP^) significantly influenced macrophage polarization *in vitro*. There was no significant difference between BM-MSCs and BM-MSCs^HYP^ in inhibiting the M1 phenotype (*p* = 0.2502), but WJ-MSCs^HYP^ were significantly more effective than WJ-MSCs (*p* = 0.0073). Notably, both WJ-MSCs and WJ-MSCs^HYP^ outperformed their BM counterparts in suppressing M1 macrophages (*p* ≤ 0.0001 for both) (Figure 2B; Table S1). Similarly, WJ-MSCs^HYP^ showed superior efficacy over BM-MSCs^HYP^ in promoting arginase-1^+^ M2 macrophages (*p* ≤ 0.0001) (Figure 2C; Table S1).

### WJ-MSCs^HYP^ enhanced IDO, PGE2, IL-10, and TGF-β secretion while inhibiting IFN-γ, TNF-α, IL-6, and IL-1β

**Figure 3:**
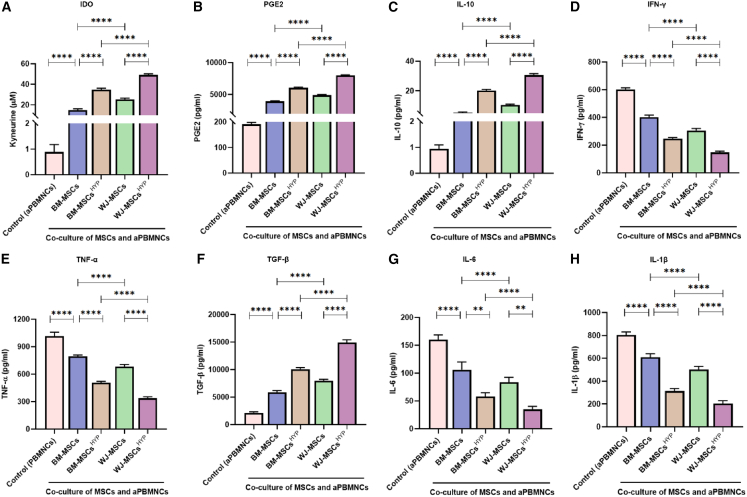
Effect of naive and hypoxia-preconditioned MSCs (MSCs, MSCs^HYP^) on the secretion of immunomodulatory molecules and cytokines (A) The bar graphs represent the relative concentration of (A) IDO (μM). (B) PGE2 (pg/mL). (C) IL-10 (pg/mL). (D) IFN-γ (pg/mL). (E) TNF-α (pg/mL). (F) TGF-β (pg/mL). (G) IL-6 (pg/mL). (H) IL-1β (pg/mL) in the direct co-culture of MSCs and aPBMNCs derived from aGVHD patients (*n* = 25). Data shown represent the mean ± SD of 25 independent experiments performed with PBMNCs derived from 25 different donors (biological replicates), with each experiment conducted in triplicate (technical replicates). Statistical analysis: Tukey’s multiple comparisons test; ∗≤0.05; ∗∗≤0.01; ∗∗∗≤0.001; ∗∗∗∗≤0.0001. BM, bone marrow; WJ, Wharton’s jelly; MSCs, mesenchymal stem cells; HYP, hypoxia-preconditioned; APO, apoptosis; aPBMNCs, activated peripheral blood mononuclear cells; IDO, indoleamine 2,3 dioxygenase; PGE2, prostaglandin E2; IL, interleukin; IFN-γ, interferon-γ; TNF-α, tumor necrosis factor-α; TGF-β, transforming growth factor β.

WJ-MSCs^HYP^ displayed a superior immunomodulatory profile compared to BM-MSCs^HYP^, with significantly higher secretion of key anti-inflammatory mediators—indoleamine 2,3 dioxygenase (IDO), PGE2, interleukin 10 (IL-10), and TGF-β and greater inhibition of pro-inflammatory cytokines and chemokines such as interferon-γ (IFN-γ), tumor necrosis factor-α (TNF-α), interleukin 6 (IL-6), and CCR6. This suggests WJ-MSCs^HYP^ are more effective at suppressing Th1- and Th17-mediated inflammatory pathways in aGVHD. While hypoxic preconditioning enhanced the immunomodulatory effects of both MSC types, the improvement was more pronounced in WJ-MSCs^HYP^ than in BM-MSCs^HYP^ (Figures 3A–3H).

### Apoptosis and efferocytosis in MSCs-mediated immunosuppression and their interaction with CD8^+^ T cell phenotypes

**Figure 4:**
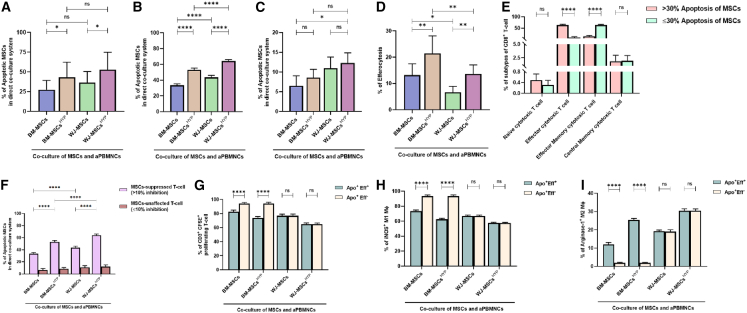
Effect of aGVHD patients-derived aPBMNCs on naive and hypoxia-preconditioned MSCs (MSCs, MSCs^HYP^) (A) The bar graph represents the percentage of apoptotic MSCs (*n* = 25). (B) The percentage of apoptotic MSCs, where apoptosis was ≥30% (*n* = 18). (C) The percentage of apoptotic MSCs, where apoptosis was <30% (*n* = 7). (D) The percentage of efferocytosis (*n* = 25). (E) The percentage of subtypes of CD8^+^ T cell (*n* = 25). (F) The percentage of apoptotic MSCs and T cell suppression (*n* = 25) in the direct co-culture of MSCs and aPBMNCs. (G) Further, the bar graph represents immunomodulation—the percentage of CD3^+^ CFSE^+^ proliferating T cell (*n* = 25). (H) The percentage of iNOS^+^ M1 macrophage (*n* = 25). (I) The percentage of arginase-1^+^ M2 macrophage (*n* = 25) under two conditions—when apoptosis of MSCs followed by their efferocytosis, and when MSCs underwent apoptosis (<30%) only in the direct co-culture of MSCs and aPBMNCs. Data shown represent the mean ± SD of 25 independent experiments performed with T cell derived from 25 different donors (biological replicates), with each experiment conducted in triplicate (technical replicates). Statistical analysis: Tukey’s multiple comparisons test; ∗≤0.05; ∗∗∗∗≤0.0001. BM, bone marrow; WJ, Wharton’s jelly; MSCs, mesenchymal stem cells; HYP, hypoxia-preconditioned; APO, apoptosis; Eff, efferocytosis; aPBMNCs, activated peripheral blood mononuclear cells.

Both MSCs and MSCs^HYP^ underwent apoptosis (Figure S2) and subsequent efferocytosis when co-cultured with aPBMNCs, with WJ-MSCs showing higher apoptosis rates than BM-MSCs, especially in samples with ≥30% apoptosis (43.48% vs. 33.42% for WJ-MSCs and 64.12% vs. 52.93% for WJ-MSCs^HYP^; *p* < 0.0001 for both) (Figures 4A–4C). BM-MSCs, however, exhibited significantly greater efferocytosis than WJ-MSCs in both naive (13.15% vs. 6.61%; *p* = 0.0129) and hypoxia-preconditioned states (21.42% vs. 13.60%; *p* = 0.0023) (Figure 4D) and in samples where either <30% or ≥30% apoptosis of MSCs and irrespective of the hypoxia conditioning (Figures S3A and S3B).

To identify immune cell subsets driving apoptosis of MSCs in aGVHD, we analyzed CD4^+^ and CD8^+^ T cell subtypes in a direct co-culture system. Our results showed that CD8^+^ T cell phenotypes were the key determinants of MSC fate. When MSCs apoptosis exceeded 30%, effector CD8^+^ T cell predominated (64.10%). Conversely, when apoptosis was ≤30%, effector memory CD8^+^ T cell were more common (64.38%) (Figure 4E). In contrast, CD4^+^ T cell subtypes showed no significant variation with MSCs’ apoptosis levels (Figure S4).

We evaluated the link between apoptosis of MSCs and their immunosuppressive capacity. Using a 10% median cutoff for CD3^+^ T cell proliferation inhibition, samples were grouped into ≤10% and >10% inhibition. Higher inhibition (>10%) was associated with increased MSCs’ apoptosis, while the ≤10% group showed lower apoptosis across treatment conditions. WJ-MSCs exhibited greater apoptosis than BM-MSCs (43.48% vs. 33.42%; *p* ≤ 0.0001), with hypoxia preconditioning further enhancing apoptosis in both (64.12% vs. 52.93%; *p* ≤ 0.0001) (Figure 4F).

We investigated whether MSC-induced immunomodulation in aGVHD depends solely on apoptosis or requires both apoptosis and efferocytosis. For BM-MSCs and BM-MSCs^HYP^, both apoptosis and efferocytosis were essential to inhibit T cell proliferation and promote M2 macrophage polarization. Conversely, in WJ-MSCs and WJ-MSCs^HYP^, apoptosis alone sufficed to suppress T cell proliferation, decrease M1 macrophages, and enhance M2 polarization (Figures 4G–4I).

### WJ-MSCs^HYP^ elevated mitochondrial transfer and modulated mitoenergetics and oxidative stress

**Figure 5:**
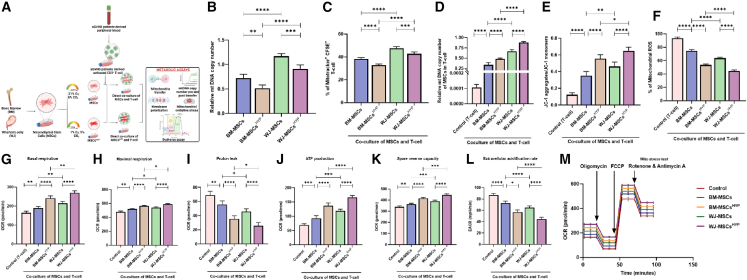
Transfer of mitochondria from MSCs, MSCs^HYP^-modulated T cell metabolism (A) Diagrammatic representation of the direct co-culture model for the assessment of modulation in T cell metabolism. (B) The bar graph represents mitochondrial (mt) DNA copy number of MSCs. (C) The percentage of Mitotracker^+^ CFSE^+^ T cell. (D) mt DNA copy number of MSCs in T cell post-24 h co-culture. (E) The ratio of JC-1 aggregates/JC-1 monomers. (F) The percentage of mitochondrial ROS. (G) Basal mitochondrial respiration. (H) Maximal respiration. (I) Proton leak. (J) ATP production. (K) Spare reserve capacity. (L) Extracellular acidification rate. (M) Real-time changes in the oxygen consumption rate of T cell with or without direct co-culture of MSCs, MSCs^HYP^. Data shown represent the mean ± SD of 25 independent experiments performed with T cell derived from 25 different donors (biological replicates), with each experiment conducted in triplicate (technical replicates). Statistical analysis: Tukey’s multiple comparisons test; ∗≤0.05; ∗∗∗∗≤0.0001. BM, bone marrow; WJ, Wharton’s jelly; MSCs, mesenchymal stem cells; HYP, hypoxia-preconditioned; mt, mitochondria; JC-1, 5,5′,6,6′-tetrachloro-1,1′,3,3′-tetraethylbenzimidazolocarbocyanine iodide; OCR, oxygen consumption rate; EACR, extracellular acidification rate.

Mitochondrial transfer from MSCs has been shown to modulate immune cell function and support immune homeostasis.^18^ To explore this in the context of aGVHD, we assessed mitochondrial transfer from MSCs to activated immune cells derived from aGVHD patients and examined its impact on immune metabolism *in vitro* (Figure 5A). We first measured mtDNA content in MSCs without co-culture. WJ-MSCs exhibited significantly higher mtDNA copy numbers than BM-MSCs (1.1684 vs. 0.722; *p* ≤ 0.0001). Hypoxia preconditioning reduced mtDNA levels in both cell types, yet WJ-MSCs^HYP^ still retained higher mtDNA than BM-MSCs^HYP^ (0.912 vs. 0.516; *p* ≤ 0.0001) (Figure 5B). Next, we assessed mitochondrial transfer from MSCs to T cell and found that WJ-MSCs transferred mitochondria more efficiently than BM-MSCs (47.63% vs. 38.26%; *p* ≤ 0.0001). Hypoxic conditioning did not hamper this enhanced transfer capacity (42.75% vs. 32.86%; *p* ≤ 0.0001) (Figures 5C and S5).

We also quantified the mtDNA transferred from MSCs to T cell following co-culture with WJ-MSCs, showing significantly higher mtDNA levels than those with BM-MSCs (0.663 vs. 0.336; *p* ≤ 0.0001), which were enhanced with hypoxia preconditioning (0.882 vs. 0.481; *p* ≤ 0.0001) (Figure 5D).

These findings suggest that mitochondrial transfer may play a role in modulating T cell function and immune metabolism. To investigate this, we assessed mitochondrial health in T cell from aGVHD patients using 5,5′,6,6′-tetrachloro-1,1′,3,3′-tetraethylbenzimidazolocarbocyanine iodide (JC-1) dye, in the presence or absence of MSCs. The T cell from aGVHD patients showed mitochondrial depolarization, indicated by a reduced JC-1 aggregates/monomers ratio, which was restored on co-culture with MSCs. Notably, WJ-MSCs induced a higher JC-1 ratio than BM-MSCs (0.462 vs. 0.352; *p* ≤ 0.001), which was enhanced with hypoxia preconditioning (0.648 vs. 0.554; *p* ≤ 0.05) (Figure 5E).

To further evaluate mitochondrial function, we measured mitochondrial reactive oxygen species (ROS) levels using MitoSOX dye. In line with the polarization data, WJ-MSCs more effectively reduced mitochondrial ROS than BM-MSCs (63.59% vs. 74.62%; *p* ≤ 0.0001), which was further enhanced with hypoxia preconditioning (44.46% vs. 53.53%; *p* ≤ 0.0001) (Figure 5F).

The impact of both MSCs and MSCs^HYP^ on T cell mitochondrial health was evaluated using the seahorse extracellular flux mitochondrial stress assay. Our findings indicated that both MSCs and MSCs^HYP^, irrespective of their tissue origin (BM or WJ), improved the basal respiration rate, maximal respiration rate, ATP production, and spare respiratory capacity, oxygen consumption rate (OCR), with a concurrent decrease in proton leak and extracellular acidification rate (EACR), highlighting the metabolic shift in T cell toward oxidative phosphorylation over glycolysis, reflecting improved mitochondrial health and function (Figures 5G–5M). Although the cumulative OCR traces of T cell grown alone and in co-culture with MSCs or MSCs^HYP^ were closely aligned across groups (Figure 5M), individual quantitative measurements of mitochondrial parameters identified striking changes. Basal respiration, maximal respiration, ATP production, and spare respiratory capacity were all modulated significantly in co-culture conditions (Figures 5G, 5H, 5J, and 5K), which suggests that MSCs affect T cell bioenergetics despite the overall similarity in OCR kinetics.

### WJ-MSCs enriched in proteins that mediate immune regulation through metabolic reprogramming

To further elucidate the underlying mechanisms contributing to superior immunomodulation capabilities of WJ-MSCs, we conducted label-free proteomics analysis on the culture-conditioned media (CCM) of WJ-MSCs alone (*n* = 3), and their co-culture with aPBMNCs (*n* = 3), BM-MSCs alone (*n* = 1), and their co-culture with aPBMNCs (*n* = 1).

Comparative analysis of WJ-MSCs in isolation against their co-culture condition revealed a total of 336 dysregulated proteins, of which 152 proteins displayed statistically significant alterations with a Log_2_-fold change threshold of ≥1.5 and ≤ −1.5. Specifically, 87 proteins were downregulated and 65 upregulated in WJ-MSCs alone-patterns that were reversed in the co-culture condition (Figures S6A–S6D).

Gene ontology (GO) analysis of proteins upregulated in WJ-MSCs revealed that 43.75% were associated with high-density lipoproteins, highlighting a strong link to lipid metabolism and transport. Additionally, 12.73% were localized to the platelet alpha granule lumen, suggesting roles in hemostasis and immune regulation, while 6.25% were linked to podosomes, implicating functions in cell adhesion and matrix degradation (Figure S7A). Further biological and molecular function analyses indicated that WJ-MSCs mediate immune regulation via diverse pathways, including complement activation and metabolic reprogramming. Notably, 30.34% of enriched proteins were involved in complement activation, with contributions from the classical pathway (18.84%), phagocytosis (5.8%), and endocytosis (1.45%) (Figures S7B and S7C).

Further analysis of upregulated proteins revealed enrichment in key immune system processes, particularly within the complement system, involving the lectin pathway (42.88%), classical pathway (28.57%), and alternative pathway (14.29%) (Figures S8A and S8B). Kyoto Encyclopedia of Genes and Genomes (KEGG) pathway analysis highlighted the role of metabolic reprogramming in immune regulation, with significant enrichment in carbohydrate metabolism (33.33%) and cholesterol metabolism (16.67%) (Figure S8C). Reactome pathway analysis reinforced these findings, showing involvement in plasma lipoprotein assembly (21.05%), carbohydrate metabolism (15.79%), and complement cascade regulation (26.32%) (Figure S8D). Cellular component analysis of downregulated proteins in WJ-MSCs alone showed that 29.41% localized to the secretory granule lumen, highlighting their role in secreting bioactive molecules. Additionally, 11.76% of proteins were associated with granule lumen, cortical actin cytoskeleton, focal adhesions, and melanosomes, structures important for cellular signaling and integrity (Figure S9A).

Biological process analysis revealed significant enrichment for nucleoside diphosphate phosphorylation (35.59%), along with NADH regeneration (16.69%) and protein refolding (10.17%), suggesting involvement in stress responses mediated by heat shock and histone proteins (Figure S9B).

Molecular function analysis highlighted notable enrichment in MHC class II protein complex binding (10.53%) and phospholipase A2 inhibitor activity (41.92%), both critical for immune regulation. Additional pathways included the intrinsic apoptotic signaling pathway (1.69%), actin filament reorganization (12.04%), and cadherin binding (5.26%) (Figure S9C).

Functional enrichment of downregulated proteins further revealed insights into immunomodulation and metabolic reprogramming. Notably, 50% of these proteins were involved in the antimicrobial humoral immune response, particularly antimicrobial peptides (Figures S10A and S10B).

KEGG pathway analysis showed significant involvement in systemic lupus erythematosus (SLE) (37.5%), gluconeogenesis (12.5%), and antigen processing and presentation (12.5%) (Figure S10C).

Meanwhile, Reactome pathway analysis highlighted a strong association with activation of RHO GTPases, activating Protein Kinase N (PKN) (83.52%), which regulate cell growth and differentiation. Downregulated pathways also included gluconeogenesis (3.3%) and Janus Kinase-Signal Transducer and Activator of Transcription (JAK-STAT) signaling following IL-12 stimulation (3.3%) (Figure S10D).

Comparative analysis of BM-MSCs alone (*n* = 1) versus their co-culture with aPBMNCs (*n* = 1) identified 339 dysregulated proteins with statistically significant changes (Log_2_-fold change ≥1.5 or ≤ −1.5). Of these, 309 proteins were upregulated and 65 downregulated in the co-culture condition, with the opposite pattern observed in BM-MSCs alone (Figures S11A and S11B).

GO analysis of upregulated proteins in the co-culture revealed a shift in the BM-MSC secretory profile toward tissue repair and regeneration, marked by enrichment in proteins involved in cytoskeleton remodeling (33.74%), integrin-mediated pathways (2.15%), and wound healing (3.76%). Additionally, proteins linked to oxidative stress protection, such as antioxidant activity (4.84%) and thioredoxin peroxidase (3.45%), as well as those involved in proteasome complex formation (5.49%), were enriched (Figures S12A–S12C).

Unlike WJ-MSCs, BM-MSCs showed limited immune regulatory involvement, suggesting their primary role in tissue repair and oxidative stress defense, with comparatively weaker immunomodulatory capacity.

Functional analysis further indicated enrichment of proteins related to JAK-STAT signaling via IL-12 stimulation (6.25%) and neutrophil degranulation and apoptosis (15.78%). However, a significant presence of proteins linked to viral carcinogenesis (40%) and infections by *Salmonella* Typhi (10%) and *E. coli* (6.67%) was also noted, implying a potential susceptibility of BM-MSCs to secondary infections and limiting their immune modulatory effectiveness compared to WJ-MSCs (Figures S13A–S13D).

## Discussion

This study investigates how hypoxia preconditioning of MSCs, which simulates their natural microenvironment, influences the immunomodulatory and metabolic functions of MSCs derived from BM and WJ. By combining functional assays with proteomic analysis, we sought to elucidate the mechanisms by which MSCs regulate immune responses in aGVHD.

Our study showed that hypoxia-preconditioned MSCs from both BM and WJ maintained their fibroblast-like morphology, surface markers, trilineage differentiation capacity, and chromosomal stability, aligning with previous reports.^6^^,^^10^^,^^19^^,^^20^^,^^21^^,^^22^^,^^23^^,^^24^^,^^25^ Although some studies noted altered differentiation potentials under hypoxia,^26^^,^^27^ our results confirm the preservation of MSCs’ parental characteristics after hypoxia preconditioning.

Our findings demonstrate that 1% O_2_ significantly enhances the immunoregulatory function of MSCs by reducing CD3^+^ T cell proliferation, promoting Tregs induction, and favoring Th2/Th9 polarization while suppressing Th1 and Th17 responses, as reported previously.^6^^,^^28^^,^^29^ Additionally, 1% O_2_ selectively decreases αβ CD4^+^ T cell, the key drivers of aGVHD, and increases the γδ T cell subset, which mitigates aGVHD. The reduction in αβ and Th17 cells correlated with greater regulatory T cell differentiation, consistent with previous studies.^30^^,^^31^ Notably, WJ-MSCs^HYP^ demonstrated superior immunomodulatory effects compared to BM-MSCs^HYP^ *in vitro*, highlighting their potential to sustain immune tolerance and foster an environment conducive to humoral immunity and tissue repair.^6^

Both MSCs and MSCs^HYP^ significantly promoted macrophage polarization *in vitro*, aligning with previous findings.^6^ Notably, WJ-MSCs^HYP^ induced a higher proportion of arginase-1^+^ M2 macrophages compared to BM-MSCs^HYP^, while both WJ-MSCs and WJ-MSCs^HYP^ more effectively suppressed the M1 phenotype than their BM-derived counterparts.

Previous studies show that MSCs exert immunoregulatory effects mainly through paracrine signaling.^32^^,^^33^^,^^34^ Consistent with this, exposure to 1% O_2_ increased immunosuppressive factors (IDO, PGE2, IL-10, and TGF-β) and reduced pro-inflammatory cytokines (IL-6, IFN-γ, TNF-α, and IL-1β). Elevated IDO and PGE2 supported Treg differentiation and suppressed Th1/Th17 responses by downregulation of IL-6 and IL-1β, while IL-10 and TGF-β promoted Treg and M2 macrophage polarization. Reduced IFN-γ and TNF-α further inhibited M1 macrophage activation, reinforcing the anti-inflammatory profile.

MSCs mediate immunoregulation, largely through apoptosis and subsequent efferocytosis, which drive anti-inflammatory responses. BM-MSCs, both naive and hypoxia preconditioned, utilized this mechanism to suppress T cell proliferation and promote M2 macrophage polarization. In contrast, WJ-MSCs, despite exhibiting higher apoptosis and reduced efferocytosis, are consistent with our previous observations,^6^ displayed superior immunosuppressive activity, indicating reliance on soluble mediators or cell-cell interactions rather than apoptotic clearance (efferocytosis). Proteomic profiling further showed downregulation of complement proteins and galectin-1 (LGALS1) in WJ-MSCs co-cultures with aPBMNCs, which implies a reduced availability of opsonic bridges and pro-resolving signals that facilitate efferocytosis and warrant validation through functional gain- and loss-of-function assays.^35^^,^^36^^,^^37^ Mitochondria are central to MSCs-mediated immunoregulation, enhancing immune cell metabolism, promoting anti-inflammatory responses, and restoring mitochondrial function via intercellular transfer.^38^^,^^39^^,^^40^ We observed higher mtDNA content in WJ-MSCs compared to BM-MSCs, consistent with greater metabolic activity^41^ and immunoregulatory potential.^42^ Hypoxia reduced mtDNA levels but did not impair transfer efficiency, with >95% of T cell acquiring MSCs-derived mitochondria. Although the exact mode of transfer (tunneling nanotubes and/or extracellular vesicles [EVs]) remains unresolved, WJ-MSCs^HYP^ more effectively restored mitochondrial polarization, reduced ROS, and improved T cell bioenergetics (increased basal and maximal respiration, ATP production, and spare respiratory capacity) with improved mitochondrial efficiency (reduced proton leak and EACR), consistent with enhanced metabolic support under hypoxia.^13^^,^^38^^,^^40^ While we used JC-1 to assess membrane potential, validation with Tetramethylrhodamine, ethyl ester/Tetramethylrhodamine methyl ester (TMRE/TMRM) is warranted. Importantly, *in vivo* evidence from lung injury models shows rapid mitochondrial transfer from MSCs to macrophages, augmenting their phagocytic function,^43^^,^^44^ supporting physiological relevance, but additional *in vivo* experiments are needed to prove this mechanism in the GVHD model. Together, our findings show that WJ-MSCs predominantly mediate immunoregulation through mitochondrial transfer and apoptosis, whereas BM-MSCs rely on apoptosis coupled with efferocytosis, underscoring distinct source-specific mechanisms in aGVHD therapy. The degree of MSCs apoptosis correlated with enhanced suppression of CD3^+^ T cell proliferation, suggesting that apoptosis is not merely a bystander effect of cytotoxic T lymphocyte (CTL) activity but rather an integral mechanism amplifying MSC-mediated immunoregulation. Importantly, distinct CTL subsets exhibited divergent effects: effector CTLs induced rapid and extensive apoptosis, thereby potentiating immunosuppression, while effector memory CTLs engaged MSCs less aggressively, resulting in moderate cytotoxicity and correspondingly reduced immunomodulation. These findings highlight the dynamic crosstalk between MSCs and specific CTL subsets, underscoring the role of effector CTL-driven apoptosis as a pivotal pathway shaping MSCs’ immunoregulatory outcomes.

In this study, we demonstrate that hypoxia-preconditioned MSCs, particularly those derived from WJ, exhibit enhanced immunomodulatory and metabolic regulatory functions compared to BM-MSCs. Through a combination of functional assays, mitochondrial analyses, and proteomic profiling, we revealed that MSCs^HYP^-mediated effects on immune cell behavior are closely tied to their capacity for mitochondrial transfer, metabolic reprogramming, and paracrine signaling. These findings provide mechanistic insight into the superior therapeutic potential of WJ-MSCs^HYP^ in inflammatory disorders such as aGVHD, underscoring the importance of cell source and preconditioning strategies in optimizing MSCs-based immunotherapy.

### Limitations of the study

Despite providing key insights into the immunomodulatory functions of hypoxia-preconditioned MSCs, this study has several limitations. The reliance on *in vitro* models may not fully capture the complexity of the aGVHD microenvironment, donor variability could affect reproducibility, and the focus on selected immune subsets excluded relevant populations such as dendritic cells and NK cells. Proteomic findings remain correlative and require functional validation, and *in vivo* studies are needed to confirm mechanistic relevance. Lastly, large-scale good manufacturing practice (GMP) translation of hypoxia preconditioning is challenged by difficulties in maintaining stable oxygen regulation and uniform exposure in bioreactors, which are designed for normoxia.^45^ Emerging solutions, including perfusion-based and hollow-fiber bioreactors with integrated oxygen control,^46^^,^^47^ and pharmacological mimetics (e.g., prolyl hydroxylase inhibitors) may help overcome these barriers.^48^^,^^49^^,^^50^ Collectively, these findings highlight both the promise and the translational hurdles of hypoxia-preconditioned MSCs in managing aGVHD.

In conclusion, our study highlights that hypoxia preconditioning enhances the immunoregulatory capacity of MSCs, with WJ-MSCs^HYP^ demonstrating superior efficacy over BM-MSCs^HYP^. WJ-MSCs mediate immune suppression primarily through apoptosis, soluble factor secretion, and mitochondrial transfer, independent of efferocytosis. In contrast, BM-MSCs rely on both apoptosis and efferocytosis to exert immunomodulatory effects. These source-specific mechanisms are supported by distinct proteomic and metabolic profiles. Our findings underscore the therapeutic promise of WJ-MSCs^HYP^ for immune-mediated conditions such as aGVHD.

## Resource availability

### Lead contact

Further information and requests for resources should be directed to and will be fulfilled by the lead contact, Prof. Ranjit Kumar Sahoo (drranjitmd@gmail.com).

### Materials availability

This study did not generate new, unique reagents.

### Data and code availability

• Proteomics data have been deposited in the PRIDE archive repository and are available via ProteomeXchange with identifier PXD074405. All other data supporting the findings of this study are available from the corresponding author upon reasonable request.
• No custom code was generated in this study. Proteomics data were analyzed using publicly available software.
• No unique reagents were generated in this study. Any additional information required to reanalyze the data reported in this paper is available from the lead contact upon request.

## Acknowledgments

The authors express their gratitude to the All India Institute of Medical Sciences (AIIMS), New Delhi, India, for facilitating the execution of the study. Schematic representative figures illustrating the methodology were created using Biorender.com. The study has been supported by the 10.13039/501100001411Indian Council of Medical Research, New Delhi, India (grant no. 2021/14763).

## Author contributions

Mohini Mendiratta performed the experiments, acquired, analyzed, and interpreted the data, and wrote the manuscript; Meenakshi Mendiratta and V.S. were involved in performing experiments and data interpretation, and analysis; S.M. was involved in data interpretation and analysis and provided resources; L.M. was involved in interpreting proteomics results and drafted the proteomics section of the manuscript; H.P., S.R., and R.G. contributed to data interpretation and analysis; S. Bandyopadhyay and G.H. were involved in the interpretation of proteomics data; S. Bakhshi, V.D., D.P., M.A., A.K.G., P.S.M., R.P.,M. Mahapatra, T.S., and R.D. provided patient samples and their clinical details; B.N., T.D.S., S.K., R.A.M., and S.K.S. contributed to data interpretation and analysis; R.K.S. conceptualized the study, provided resources, designed and supervised the experiments, interpreted the data, and reviewed and edited the manuscript. All the authors critically reviewed and approved the final version of the manuscript.

## Declaration of interests

The authors declare no competing interests.

## STAR★Methods

### Key resources table

**Table:** 

REAGENT or RESOURCE	SOURCE	IDENTIFIER
**Antibodies**		

Rabbit polyclonal anti-cleaved caspase-3	Cell Signaling Technology	Cat. # 9661S
Anti-human CD105 (PE, clone TEA3/17.1.1)	Beckman Coulter	Cat. # B76299
Anti-human CD73 (RB613, clone AD2)	BD OptiBuild ^TM^	Cat. # 758623
Anti-human CD90 (APC-A750, clone Thy-1/310)	Beckman Coulter	Cat. # B36121
Anti-human CD29 (FITC, clone K20)	Beckman Coulter	Cat. # IM0791U
Anti-human HLA-I (RY775, clone W6/32)	BD OptiBuild ^TM^	Cat. # 770875
Anti-human HLA-II (PB, clone Immu-357)	Beckman Coulter	Cat. # B36291
Anti-human CD34 (PC5 clone 581)	Beckman Coulter	Cat. # IM2648U
Anti-human CD45 (KrO, clone J33)	Beckman Coulter	Cat. # A96416
Anti-human Annexin-V (APC)	BD Pharmingen^TM^	Cat. # 567356, RRID: AB_2868885
Anti-human 7AAD	BD Pharmingen^TM^	Cat. # 559925
CFSE	BD Horizon^TM^	Cat. # 565082
Anti-human CD3 (PC5.5, clone UCHT1)	Beckman Coulter	Cat. # A66327
Anti-human CD4 (ECD, clone SFCI1T4D11)	Beckman Coulter	Cat. # 6604727
Anti-human CD4 (PB, clone 13B8.2)	Beckman Coulter	Cat. # B49197
Anti-human CD8 (APC-Alexa Fluor 750, clone B9.11)	Beckman Coulter	Cat. # A94686
Anti-human CD25 (APC-Alexa Flour 700, clone B1.49.9)	Beckman Coulter	Cat. # A86356
Anti-human FOXP3 (PE, clone 259D/C7)	BD Pharmingen^TM^	Cat. # 560082
Anti-human CD45RA (ECD, clone 2H4LDH11LDB9)	Beckman Coulter	Cat. # IM2711U
Anti-human CXCR3 (BV421, clone IC6)	BD Horizon^TM^	Cat. # 562558
Anti-human CXCR5 (Per-CP Cy5.5, clone RF8B2)	BD Pharmingen^TM^	Cat. # 562781
Anti-human CCR10 (PE, clone 1B5)	BD Pharmingen^TM^	Cat. # 563656
Anti-human CCR4 (PE-Cy7, clone 1G1)	BD Pharmingen^TM^	Cat. # 561034
Anti-human CCR7 (FITC, clone 150503)	BD Pharmingen^TM^	Cat. # 561271
Anti-human CCR6 (APC, clone 11A9)	BD Pharmingen^TM^	Cat. # 560619
Anti-human αβ (APC, clone IP26A)	Beckman Coulter	Cat. # A39500
Anti-human γδ (PE, clone IMMU510)	Beckman Coulter	Cat. # C76829
Anti-human CD68 (PE, clone Y1/82A)	Beckman Coulter	Cat. # 556078
Anti-human iNOS (FITC, clone 4E5)	Novus Biologicals	Cat. # NBP2-22119F
Anti-human Arginase-1 (APC, clone 14D2C43)	Biolegend	Cat. # 369706

**Biological samples**		

Peripheral blood mononuclear cells	Acute Graft-versus-Host-Disease patients, All India Institute of Medical Sciences	Obtained with informed consent, Institutional Human Ethics Committee approval (Ref. No.: IECPG-542/23.09.2020)
Mesenchymal Stem Cells (Bone Marrow, Wharton’s Jelly)	Healthy donors, All India Institute of Medical Sciences	Obtained with informed consent, Institutional Human Ethics Committee approval (Ref. No.: IECPG-542/23.09.2020); Institutional Committee approved for Stem Cell Research [Ref. No.: IC-SCR/110/20(R)]

**Chemicals, peptides, and recombinant proteins**		

DMEM powder, low glucose, pyruvate	Thermo Fisher Scientific	Cat. # 31600083
RPMI-1640 media	Thermo Fisher Scientific	Cat. # 11875093
Fetal Bovine Serum (FBS)	Thermo Fisher Scientific	Cat. # 10270106
Antibiotic-antimycotic	Thermo Fisher Scientific	Cat. # 15240062
Stem Pro^TM^ MSC SFM	Thermo Fisher Scientific	Cat. # A1033201
Trypsin-EDTA (0.25%)	Thermo Fisher Scientific	Cat. # 25200072
Histopaque-1077	Sigma-Aldrich	Cat. # 10771
Staurosporine from *Streptomyces* sp	Sigma-Aldrich	Cat. # S5921
Alizarin Red S	HiMedia	Cat. # GRM894
Formalin solution, neutral buffered, 10%	Sigma-Aldrich	Cat. # HT501128
Oil Red O	HiMedia	Cat. # TC256
Paraformaldehyde	Sigma-Aldrich	Cat. # 158127
Isopropanol pure, 99%	Sisco Research Laboratories	Cat. # 67800
Glacial acetic acid	Sisco Research Laboratories	Cat. # 93602
Alcian blue 8GX	HiMedia	Cat. # TC359
Indomethacin	HiMedia	Cat. # TC465
Dexamethasone	Sigma-Aldrich	Cat. # D4902
3-isobutyl-1-methylxanthine	Sigma-Aldrich	Cat. # I5879
Insulin	HiMedia	Cat. # RM8507
L-Ascorbic acid	HiMedia	Cat. # PCT0207
β-glycerophosphate	HiMedia	Cat. # TC463
Cochicine	HiMedia	Cat. # CMS342
KCl	HiMedia	Cat. # GRM698
Methanol	Sigma-Aldrich	Cat. # 34885
Giemsa solution	HiMedia	Cat. # S011
Mitomycin C from *Streptomyces caespitosus*	Sigma-Aldrich	Cat. # M4287
Phytohemagglutinin-M	Sigma-Aldrich	Cat. # 11082132001
IL-2 human	Sigma-Aldrich	Cat. # H7041
Granulocyte-Macrophage Colony-Stimulating Factor human	Sigma-Aldrich	Cat. # G5035
Lipopolysaccharides from *Salmonella enterica* serotype *typhimurium*	Sigma-Aldrich	Cat. # L6143
Human IFN-gamma Recombinant Protein	Thermo Fisher Scientific	Cat. # PHC4031
Trichloroacetic acid	HiMedia	Cat. # GRM6274
Ehrlich’s reagent	HiMedia	Cat. # R005
pHrodo^TM^ Red, succinimidyl ester	Thermo Fisher Scientific	Cat. # P36600
Mitotracker Red	Thermo Fisher Scientific	Cat. # M7512
Cell tracker Green CMFDA	Thermo Fisher Scientific	Cat. # C2925
JC-1 dye	Thermo Fisher Scientific	Cat. # T3168
MitoSOX Red	Thermo Fisher Scientific	Cat. # M36008
XF DMEM media	Agilent Technologies	Cat. # 103575
XF RPMI media	Agilent Technologies	Cat. # 103576
Glucose solution	Agilent Technologies	Cat. # 103577
Pyruvate solution	Agilent Technologies	Cat. # 103578
Glutamine solution	Agilent Technologies	Cat. # 103579
TCEP	Thermo Fisher Scientific	Cat. # T2556
Iodoacetamide	HiMedia	Cat. # RM9637
Trypsin	HiMedia	Cat. # RM612
Acetonitrile	HiMedia	Cat. # AS028
Formic acid	Sigma-Aldrich	Cat. # F0507

**Critical commercial assays**		

Annexin V binding buffer, 10X concentrate	BD Biosciences	Cat. # 556454
Stem Pro^TM^ chondrogenic differentiation kit	Thermo Fisher Scientific	Cat. # A1007101
Human Fox P3 buffer set	BD Pharmingen^TM^	Cat. # 560098
Pan T-cell isolation kit, human	Miltenyi Biotec	Cat. # 130-096-535
Pan monocyte isolation kit, human	Miltenyi Biotec	Cat. # 130-096-537
Human Prostaglandin E2 ELISA kit	Thermo Fisher Scientific	Cat. # KHL1701
Human IL-10 ELISA kit	Thermo Fisher Scientific	Cat. # EHIL10
Human IFN gamma ELISA kit	Thermo Fisher Scientific	Cat. # EHIFNG
Human TNF-α ELISA kit	Thermo Fisher Scientific	Cat. # KHC3011
Human TGF beta-1 ELISA kit	Thermo Fisher Scientific	Cat. # BMS249-4
Human IL-6 ELISA kit	Thermo Fisher Scientific	Cat. # EH2IL6
Human IL-1β ELISA kit	Thermo Fisher Scientific	Cat. # BMS224-2
Agilent Seahorse XF Cell Mito Stress Test kit	Agilent Technologies	Cat. # 103015

**Oligonuclotides**		

Primer for nuclear DNA (forward primer)	Integrated DNA Technologies	5′-TCACCCACACTGTGCCCATCTAGGA-3′
Primer for nuclear DNA (reverse primer)	Integrated DNA Technologies	5′-CAGCGGAACCGCTCAT TGCCAATGG-3′
Primer for mt DNA (forward primer)	Integrated DNA Technologies	5′-CGAAAGGACAAGAGAAATAAGG-3′
Primer for mt DNA (reverse primer)	Integrated DNA Technologies	5′-CTGTAAAGTTTTAAGTTTTATGCG-3′

**Software and algorithms**		

GraphPad Prism version 8.4.2	GraphPad software	https://www.graphpad.com

**Deposited data**		

Raw data files of LC-MS/MS	PRIDE Archive	PXD074405

### Experimental model and study participant details

This study utilized exclusively human-derived samples (Homo sapiens). Bone marrow aspirates were obtained from healthy adult donors (*n* = 10; age range: 18–65 years) at the Department of Medical Oncology, Dr. B. R. Ambedkar Institute Rotary Cancer Hospital, AIIMS, New Delhi. Both male and female donors contributed samples. Umbilical cord (UC) tissues (*n* = 10) were collected from full-term deliveries (37–40 weeks of gestation) at the Department of Obstetrics and Gynaecology, AIIMS, New Delhi. All UC samples were derived from female neonates. Peripheral blood samples were collected from patients diagnosed with grade II-IV acute graft-versus-host disease (aGVHD) (n = 25) at AIIMS, New Delhi. Both male and female patients were included in the study. The study was not specifically designed or powered to evaluate sex- or gender-based differences in outcomes, and sex-disaggregated analyses were not performed. Therefore, potential sex-related effects represent a limitation to the generalizability of the findings.

The study involved human subjects, which was approved by the Institutional Human Ethics Committee at the All India Institute of Medical Sciences, New Delhi, India (Ref. No.: IECPG-542/23.09.2020). Additionally, it included the use of human MSCs, which the Institutional Committee approved for Stem Cell Research at the same institution [Ref. No.: IC-SCR/110/20(R)]. All human sample collection was performed after obtaining written informed consent from participants or legal guardians, and all procedures were conducted in accordance with the Declaration of Helsinki and guidelines and regulations approved by the ethics committee.

### Method details

#### Isolation, characterization, and hypoxia-preconditioning of human MSCs from bone marrow (BM) and Wharton’s Jelly (WJ)

Human BM aspirates were obtained from healthy donors (*n* = 10) who were recipients of allogeneic stem cell transplants at the Department of Medical Oncology, Dr. B. R. Ambedkar Institute Rotary Cancer Hospital, AIIMS, New Delhi. Additionally, human umbilical cord (UC) tissue was collected from donors (*n* = 10) in sterile transport media containing 1000 IU/mL heparin (Gland Pharma Limited, India) and 200 μg/mL gentamicin (Thermo Fisher Scientific, USA) from the Department of Obstetrics and Gynaecology at AIIMS, New Delhi. BM-MSCs and WJ-MSCs were isolated following our previously established protocol, as described elsewhere.^6^ Briefly, bone marrow aspirates were plated in 60-mm culture dishes containing low-glucose Dulbecco’s Modified Eagle’s Medium (LG-DMEM) supplemented with 10% FBS and 1% antibiotic–antimycotic solution and maintained at 37°C in a humidified incubator with 5% CO_2_ and 21% O_2_. After 72 h, the medium was replaced with LG-DMEM complete medium supplemented with StemPro™ MSC Serum-Free Medium (3:1 ratio), and medium changes were performed every third day until the cells reached approximately 80% confluency. For WJ- MSCs, a standard explant culture method was employed, wherein approximately 2 × 2 cm tissue explants were placed in 35-mm culture dishes, secured with coverslips, and overlaid dropwise with LG-DMEM complete medium. After allowing 2–3 h for attachment under standard incubation conditions (37°C, 5% CO_2_), LG-DMEM supplemented with StemPro™ MSC Serum-Free Medium (3:1 ratio) was added. At approximately 80% confluency, adherent cells were detached using 0.05% trypsin-EDTA, harvested, and passaged for further expansion and subsequent experiments.

Both BM-MSCs and WJ-MSCs (Passage-3) were characterized for their plastic adherence, surface marker profile, and trilineage differentiation potential according to the International Society for Cellular Therapy (ISCT) guidelines^51^ using our established protocols.^6^ Passages 3–5 were used for subsequent *in vitro* experiments, and these were pooled together at their respective passages. All MSCs cultures were maintained under aseptic conditions and were confirmed to be free from mycoplasma, bacterial, and fungal contamination.

MSCs were preconditioned with 1% O_2_ in 1X LG-DMEM complete media (Thermo Fisher Scientific, USA) for 24 h in a tri-gas incubator, termed hypoxia-preconditioned MSCs (MSCs^HYP^).^6^ Exposure to hypoxia (1% O_2_ for 24 h) was chosen based on our prior investigations, which showed that this duration and concentration of hypoxia optimally stabilize HIF-1α and induce the immunomodulatory mediators maximally.^6^^,^^52^

#### Karyotyping of MSCs

The cells were first expanded in culture to a sufficient number, typically 2∗10^6^ cells/ml, in 1X LG-DMEM complete medium (Thermo Fisher Scientific, USA) at 37°C, 5% CO_2_. To synchronize the cells in metaphase, they were treated with colchicine (0.1 μg/mL) (Thermo Fisher Scientific, USA) for 6 h. After colchicine treatment, the cells were harvested using 0.05% Trypsin-EDTA (Thermo Fisher Scientific, USA), centrifuged at 800 rpm for 5 min, and resuspended in a hypotonic solution of 0.075 M KCl (Sisco Research Laboratories Pvt. Ltd., India) for 30 min at 37°C. After centrifugation, the cells were fixed by adding fixative [methanol (Sisco Research Laboratories Pvt. Ltd., India): acetic acid (Sisco Research Laboratories Pvt. Ltd., India) in a 3:1 ratio] dropwise, incubated for 10 min, and this process was repeated 2–3 times. Microscope slides were prepared by dropping the fixed cell suspension onto clean slides and allowing them to air dry. The slides were stained with Giemsa solution (Sisco Research Laboratories Pvt. Ltd., India) at 1:5 dilution for 15 min, rinsed with distilled water, and air dried. The stained slides were observed under a light microscope (Zeiss Axio Imager 2, Zeiss, Germany) at 100x magnification to visualize the metaphase chromosomes. The slides were scanned, images were captured using Metafer software (MetaSystems, Germany), and the resulting karyotype was generated using IKAROS software (MetaSystems, Germany).^53^

#### *In vitro* T-cell proliferation assay

Peripheral blood (PB) was collected from grade II-IV aGVHD patients (*n* = 25) in sterile sodium heparin-coated vacutainers (BD Biosciences, US). CD3^+^ T cell was isolated using the Pan T cell isolation kit (Miltenyi Biotec, USA) through negative selection and cultured for 48 h in the presence of PHA (1 μg/ml) (Sigma, USA) and IL-2 (50IU/ml) (Thermo Fisher Scientific, USA), following our previously established protocols described elsewhere.^6^

T cell proliferation was evaluated using our previously established protocol^6^ in a direct 2D co-culture setup, where mitomycin-treated BM-MSCs or WJ-MSCs (MSCs, MSCs^HYP^) were cultured with CFSE-labeled activated CD3^+^ T cell at a 1:10 ratio for 72 h.

#### Induction of regulatory T cell (Tregs)

The induction of Tregs was assessed in the direct co-culture of BM-MSCs or WJ-MSCs (MSCs, MSCs^HYP^) and activated CD3^+^ T cell at a 1:10 ratio for 5 days using established protocols.^6^^,^^54^

#### Enumeration of effector memory helper T cell subtypes (Th1, Th2, Th9, and Th17)

The proportions of Th1, Th2, Th9, and Th17 cells were quantified in the 5-day co-culture of mitomycin-treated MSCs (MSCs, MSCs^HYP^) and activated T cell by staining with fluorochrome-conjugated anti-human monoclonal antibodies specific for CXCR3, CXCR5, CCR10, CCR4, CCR7, and CCR6 (BD Biosciences, USA) at 37°C for 30 minutes. This was followed by additional staining with anti-human fluorochrome-conjugated CD3, CD4, CD8, and CD45RA monoclonal antibodies. A minimum of 50,000 cells was acquired using a DxFlex flow cytometer (Beckman Coulter, USA) and the data were analyzed using Kaluza software version 2.1 (Beckman Coulter, USA). Activated T-cell cultured without MSCs served as a control to establish baseline expression levels of Th1, Th2, and Th17.^6^

#### Enumeration of αβ and γδ CD4^+^ T-cell

The proportions of αβ and γδ CD4^+^ T-cell was quantified in the 3-day co-culture of mitomycin-treated MSCs (MSCs, MSCs^HYP^) and activated T-cell by staining with fluorochrome-conjugated anti-human monoclonal antibodies specific for CD3, CD4, CD8, αβ, and γδ monoclonal antibodies for 30 minutes in the dark. A minimum of 50,000 cells was acquired using a DxFlex flow cytometer (Beckman Coulter, USA) and the data were analyzed using Kaluza software version 2.1 (Beckman Coulter, USA). Activated T-cell cultured without MSCs served as a control to establish baseline expression levels of αβ and γδ T-cell.^55^

#### Macrophage polarization

CD14^+^ monocytes were isolated from PBMNCs using a pan-monocyte isolation kit (Miltenyi Biotec, USA), and macrophage polarization was assessed in the direct co-culture of mitomycin-treated BM-MSCs or WJ-MSCs (MSCs, MSCs^HYP^) and M1 macrophages at a ratio of 1:10 for 3 days as described elsewhere.^6^

#### Estimation of secreted cytokines, chemokines, and other soluble factors

The concentration of various secreted factors, including PGE2, Interleukin-10 (IL-10), Interferon-γ (IFN-γ), Tumor necrosis factor-α (TNF-α), TGF-β, Interleukin-6 (IL-6), and Interleukin-1β (IL-1β) (Thermo Fisher Scientific, USA) was quantified in the culture-conditioned media (CCM) collected after a 3-day co-culture of mitomycin-treated MSCs and aPBMNCs using ELISA with a PR4100 microplate reader (Bio-Rad, USA) following manufacturer’s instructions. CCM from the culture of aPBMNCs alone (without co-culture) served as a control for baseline expression of secreted soluble factors.^56^

#### Kynurenine assay

The kynurenine assay evaluated the IDO activity in the direct co-culture of MSCs (MSCs, MSCs^HYP^) and aPBMNCs. Following 3 days of co-culture, 200 μl of the culture supernatant was collected from each well and deproteinized by adding an equal volume of 30% trichloroacetic acid (TCA) (Sigma-Aldrich, Saint Louis, MO, USA). The mixture was vortexed and centrifuged at 10,000 × g for 10 minutes at 4°C, and the clear supernatant was transferred to a new Eppendorf tube (Genaxy Scientific, India). For kynurenine detection, 100 μl of the deproteinized supernatant was mixed with an equal volume of Ehrlich’s reagent (Sigma-Aldrich, Saint Louis, MO, USA) in a 96-well plate (Genaxy Scientific, India) and incubated at room temperature for 10 minutes to develop a yellow color. The absorbance was measured at 490 nm using a PR4100 microplate reader (Bio-Rad, USA), and kynurenine levels were quantified by interpolating the absorbance values from a standard curve prepared using known concentrations of kynurenine. CCM from the culture of aPBMNCs alone (without co-culture) served as a control for the baseline expression of secreted IDO.^57^

#### Annexin-V/7AAD assay

The proportion of apoptosis of MSCs was assessed in the 3-day co-culture of mitomycin-treated MSCs and activated T-cell using Annexin-V/7AAD (BD Biosciences, USA) staining to assess the effect of T-cell on MSCs.^6^

#### *In vitro* MSCs phagocytosis assay

CFSE-labeled aPBMNCs were treated with pH Rhodo red succinimidyl ester-labeled MSCs (Thermo Fisher Scientific, USA) at a 1:10 ratio for 72 hours. The percentage of CD14^+^ monocytes that were CFSE-positive also exhibited the pH Rhodo red signal, which was utilized to calculate the engulfed MSCs by the monocytes using a DxFlex flow cytometer (Beckman Coulter, USA).^6^

#### T-cell phenotype assay

The subtypes of CD4^+^ and CD8^+^ T-cell were assessed in the 3-day co-culture of mitomycin-treated MSCs and aPBMNCs by staining the cell suspension with fluorochrome-conjugated anti-human CCR7 (Beckmann Coulter, USA) monoclonal antibodies at 37֯C for 30 minutes, followed by surface staining with anti-human CD3, CD4, CD8, and CD45RA (Beckmann Coulter, USA) monoclonal antibodies for 30 minutes at room temperature. A minimum of 50,000 cells were acquired using a DxFlex flow cytometer (Beckman Coulter, USA) and the data were analyzed using Kaluza software version 2.1 (Beckman Coulter, USA). Activated T-cell cultured without MSCs served as a control to establish baseline expression levels of subtypes of CD8^+^ T-cell.^58^

#### Mitochondria transfer assay

The transfer of mitochondria from MSCs to CD3^+^ T-cell was assessed in the direct co-culture of mitomycin-treated MSCs and aPBMNCs. Briefly, MSCs were pre-labeled with Mitotracker^TM^ Red (Thermo Fisher Scientific, USA) at a concentration of 100nM, and aPBMNCs were pre-labeled with Cell Tracker^TM^ Green (Thermo Fisher Scientific, USA) at a concentration of 1μM at 37֯C for 20 minutes, followed by their direct co-culture for 24 hours. The proportion of Mitotracker^+^ Cell Tracker Green^+^ cells was enumerated to assess the transfer of mitochondria from MSCs to CD3^+^ T-cell using a DxFlex flow cytometer (Beckmann Coulter, USA).^59^

#### Quantification of mitochondrial DNA (mtDNA) copy number

Genomic and mitochondrial DNA were isolated from MSCs and MSCs^HYP^ before co-culture, as well as from T-cell before and after co-culture for the assessment of mtDNA copy number using a QIAamp DNA Mini Kit (Qiagen, Netherlands), according to the manufacturer’s instructions. Real-time PCR was performed with 50 ng of DNA, 5 μl of Kappa SYBR Master Mix, and primers at a final concentration of 0.3 μM in a total reaction volume of 10 μl using a CFX96 Real-Time System (Bio-Rad). The copy number of mtDNA and nuclear DNA was quantified using the threshold cycle (Ct) values. The Delta Ct (ΔCt) was determined using the equation: Ct (mitochondrial gene) − Ct (nuclear gene), and the relative mtDNA copy number was calculated using the 2−ΔΔCt method.^59^

#### Measurement of mitochondrial membrane potential

The mitochondrial health of CD3^+^ T-cell was assessed in the 1-day co-culture of mitomycin-treated MSCs and CD3^+^ T-cell by staining the cell suspension with JC-1 dye (Thermo Fisher Scientific, USA) at a concentration of 2μM at 37֯C for 20 minutes in the dark. Subsequently, the cells were washed with 1XPBS and stained with fluorochrome-conjugated anti-human CD3 monoclonal antibody. JC-1 aggregates/JC-1 monomers ratio gated on CD3^+^ T-cell was enumerated to assess their mitochondrial health status.^60^

#### Mitochondrial Reactive oxygen Species (ROS) assay

The mitochondrial ROS level of CD3^+^ T-cell was assessed in the 1-day co-culture of CD3^+^ T-cell and mitomycin-treated MSCs by staining the cell suspension with MitoSOX Red (Thermo Fisher Scientific, USA) at a concentration of 5μM at 37֯C for 20 minutes. Subsequently, the cells were washed with 1XPBS and stained with fluorochrome-conjugated anti-human CD3 monoclonal antibody. The percentage of CD3^+^ MitoSox Red^+^ cells was quantified to enumerate the mitochondrial ROS level in CD3^+^ T-cell using a DxFlex flow cytometer (Beckmann Coulter, USA), and the data were analyzed using Kaluza Software Version 2.1. CD3^+^ T-cell cultured without MSCs served as a control to establish the baseline expression of mitochondrial ROS.^61^

#### T-cell mitochondrial bioenergetics

Oxygen consumption rate (OCR) and extracellular acidification rate (ECAR) were assessed using the Seahorse XFe24 Extracellular Flux Analyzer (Agilent Technologies, USA), which serves as an indicator for oxidative phosphorylation (OXPHOS) and glycolysis, respectively. Mitomycin-treated MSCs were co-cultured with CD3^+^ T-cell for 24 hours, followed by the separation of CD3^+^ T-cell from the cell suspension of the co-culture using negative selection.

Further, these co-cultured CD3^+^ T-cell and control (non-co-cultured) CD3^+^ T-cell were seeded separately at a density of 1∗10^6^ cells per well on CellTak-coated plates (Corning) in XF-Base Media (Agilent Technologies, USA), supplemented with 2.5 mM glucose, 1 mM sodium pyruvate (HiMedia, India), and 2 mM L-glutamine (HiMedia, India). Control CD3^+^ T-cell was used as a baseline for assessing T-cell mitochondrial bioenergetics. The OCR measurements were taken sequentially, starting from the basal level, followed by the addition of 1.0 mM oligomycin (Sigma-Aldrich, Saint Louis, MO, USA), 0.75 mM FCCP (fluorocarbonyl cyanide phenylhydrazone) (G-Biosciences, Saint Louis, MO, USA), and a combination of 0.5 mM rotenone (Sigma-Aldrich, Saint Louis, MO, USA) and antimycin (Sigma-Aldrich, Saint Louis, MO, USA) to evaluate changes in mitochondrial respiratory parameters. Data analysis was performed using Wave 2.6.1 and GraphPad Prism software.^62^

#### Identification of secreted proteins using liquid chromatography-assisted mass spectrometry (LC-MS/MS)

Protein per sample (BM-MSCs, WJ-MSCs, and their co-culture with aPBMNCs) was used for digestion and reduced with 5 mM tris(2-carboxyethyl) phosphine (TCEP) and further alkylated with 50 mM iodoacetamide and then digested with Trypsin (1:50, Trypsin/lysate ratio) for 16 h at 37°C. Digests were cleaned using a C18 silica cartridge to remove salts and dried using a speed vacuum. The dried pellet was resuspended in buffer A (2% acetonitrile, 0.1% formic acid).

Experiments were performed on an Easy-nlc-1000 system coupled with an Orbitrap Exploris mass spectrometer. 1μg of peptide sample was loaded on C18 column 15 cm, 3.0μm Acclaim PepMap (Thermo Fisher Scientific, USA) and separated with a 0–40% gradient of buffer B (80% acetonitrile, 0.1% formic acid*)* at a flow rate of 500 nl/min and injected for MS analysis. LC gradients were run for 110 minutes. MS1 spectra were acquired in the Orbitrap (Max IT = 60ms, AGQ target = 300%; RF Lens = 70%; R=60K, mass range: 375−1500; Profile data). Dynamic exclusion was employed for 30s, excluding all charge states for a given precursor. MS2 spectra were collected for the top 20 peptides. MS2 (Max IT= 60ms, R= 15K, AGC target 100%).

All samples were processed, and the RAW files generated were analyzed with Proteome Discoverer (v2.5) against the UniProt Human database. For dual Sequest and Amanda searches, the precursor and fragment mass tolerances were set at 10 ppm and 0.02 Da, respectively. The protease used to generate peptides, i.e., enzyme specificity, was set for trypsin/P (cleavage at the C terminus of “K/R: unless followed by “P”). Carbamidomethyl on cysteine as fixed modification and oxidation of methionine and N-terminal acetylation were considered as variable modifications for database search. Both peptide spectrum match and protein false discovery rate were set to 0.01 FDR.^63^

### Quantification and statistical analysis

All statistical analyses were performed using GraphPad Prism (version 8.4.3; GraphPad Software, Boston, Massachusetts, USA). Comparisons among three or more groups were conducted using one-way analysis of variance (ANOVA) followed by Tukey’s multiple comparisons post hoc test, as appropriate. For comparisons between two groups, an unpaired two-tailed Student’s t-test was applied where indicated. Data are presented as mean ± standard deviation (SD). The mean was used as the measure of central tendency, and SD represents dispersion. A p-value ≤ 0.05 was considered statistically significant.

The exact value of n for each experiment is indicated in the corresponding figure legends. In this study, n represents the number of independent biological samples (e.g., individual donors or patient samples) unless otherwise specified. Technical replicates, where performed, were averaged before statistical analysis. All statistical details, including sample size (n), statistical tests used, and significance values, are provided in the figure legends and corresponding results sections.

#### Study approval

The study involved human subjects, which was approved by the Institutional Human Ethics Committee at the All India Institute of Medical Sciences, New Delhi, India (Ref. No.: IECPG-542/23.09.2020). Additionally, it included the use of human MSCs, which the Institutional Committee approved for Stem Cell Research at the same institution [Ref. No.: IC-SCR/110/20(R)]. Informed written consent was obtained from all participants, and all procedures were conducted in accordance with the guidelines and regulations approved by the ethics committee.
