# Supplementary material for: Hypoxia-induced metabolic and apoptotic reprogramming enhances immunomodulation in Wharton’s jelly mesenchymal stem cells
Source: iScience. 2026 Mar 13;29(4):115363. doi: 10.1016/j.isci.2026.115363 (PMC13089076; doi:10.1016/j.isci.2026.115363)
Supplement: Document S1. Figures S1–S13 and Tables S1 and S2 [file mmc1.pdf]

iScience, Volume *29*

Supplemental information

Hypoxia-induced metabolic and apoptotic reprogramming enhances immunomodulation in Wharton's jelly mesenchymal stem cells

Mohini Mendiratta, Meenakshi Mendiratta, Sujata Mohanty, Hridayesh Prakash, Lakshay Malhotra, Sandeep Rai, Vijaya Sarangathem, Sabyasachi Bandyopadhyay, GuruRao Hariprasad, Ritu Gupta, Sameer Bakhshi, Vatsla Dadhwal, Deepam Pushpam, Mukul Aggarwal, Aditya Kumar Gupta, Prabhat Singh Malik, Raja Pramanik, Manoranjan Mahapatra, Tulika Seth, Rishi Dhawan, Baibaswata Nayak, Thoudam Debraj Singh, Sachin Kumar, Riyaz Ahmed Mir, Surender Kumar Sharawat, and Ranjit Kumar Sahoo

Supplemental figures

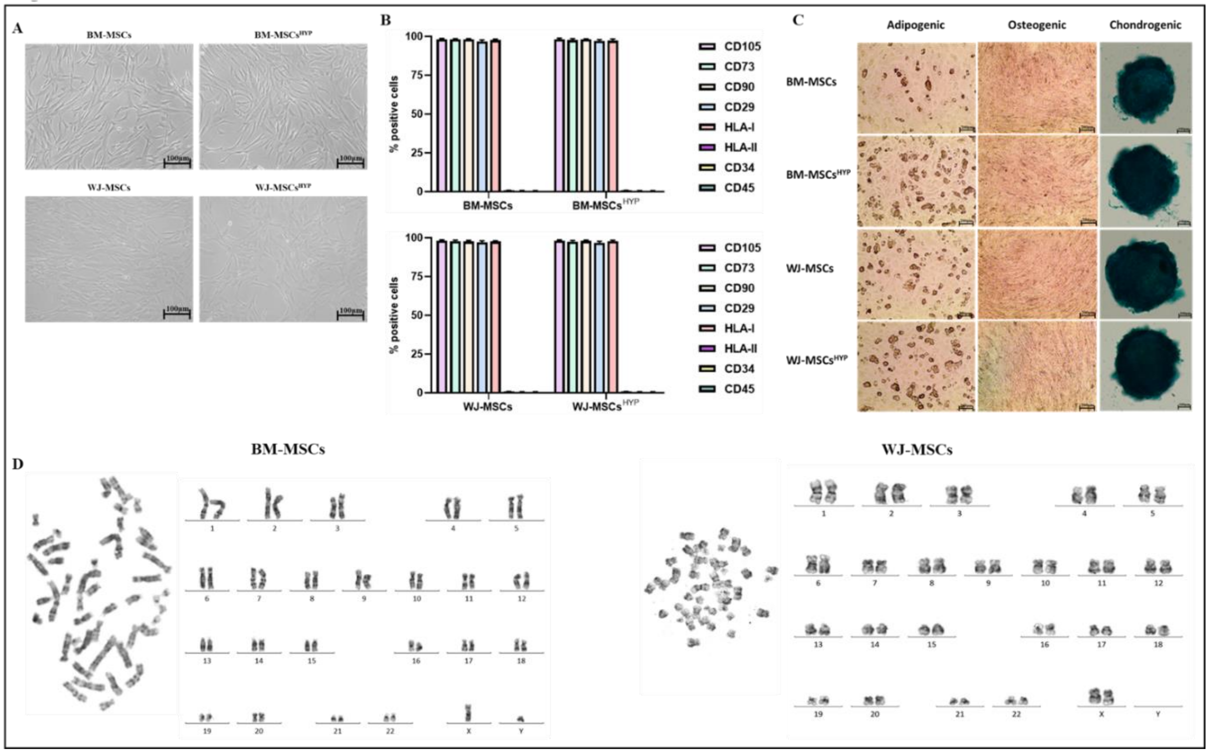

Figure S1: Characterization of tissue-specific human MSCs (BM-MSCs, BM-MSCs^{HYP}, WJ-MSCs, and WJ-MSCs^{HYP}). A) Morphological images. B) Bar graphs depict surface marker profiling using flow cytometry. C) Trilineage differentiation. Data are shown as Mean±S.D. The data shown are from independent experiments performed with MSCs derived from three different donors (biological replicates) and conducted in triplicate (technical replicates). Scale bar: 10X = 100 µm. *Abbreviations: BM: Bone marrow; WJ: Wharton's Jelly; MSCs: Mesenchymal Stem Cells; HYP: Hypoxia-preconditioned*

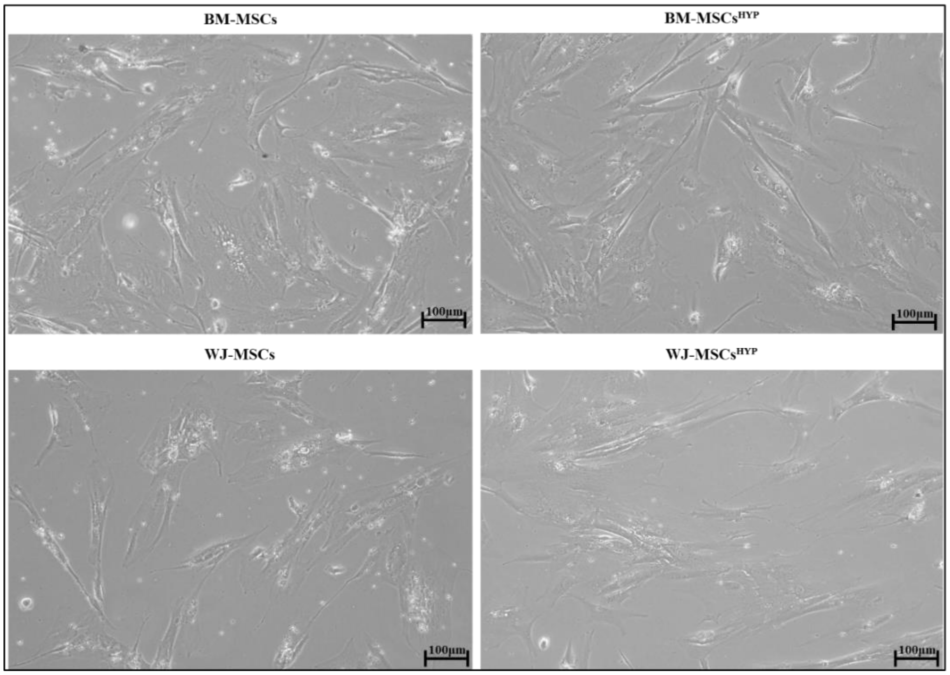

Figure S2: Representative morphological images of direct co-culture of tissue-specific human MSCs (BM-MSCs, BM-MSCs^{HYP}, WJ-MSCs, and WJ-MSCs^{HYP}) and aGVHD patients-derived T-cell. Scale bar: 10X = 100 µm. *Abbreviations: BM: Bone marrow; WJ: Wharton's Jelly; MSCs: Mesenchymal Stem Cells; HYP: Hypoxia-preconditioned*

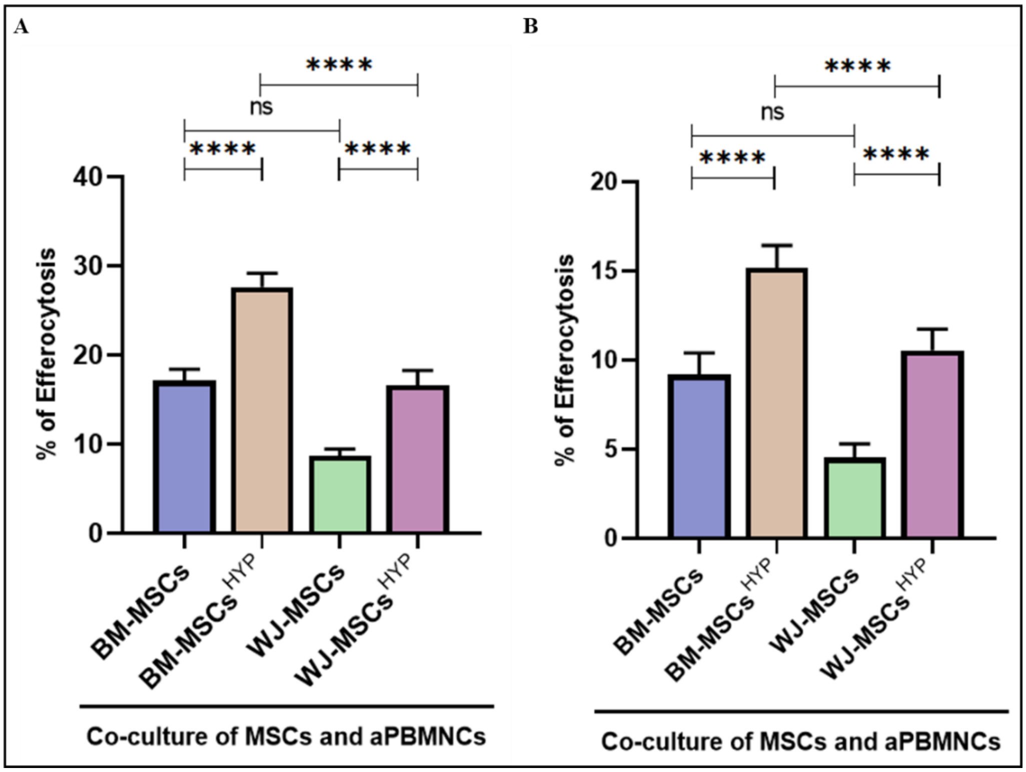

Figure S3: Efferocytosis of MSCs in the direct co-culture of aPBMNCs derived from aGVHD patient and MSCs (BM-MSCs, BM-MSCs^{HYP}, WJ-MSCs, and WJ-MSCs^{HYP}). The bar graphs show the percentage of efferocytosis stratified based on MSC apoptotic levels: A) ≥30% apoptosis (n = 18). and B) <30% apoptosis (n = 7). Data are presented as mean ± SD. In A), n represents 18 independent biological experiments performed using T cells derived from 18 different donors; in B), n represents 7 independent biological experiments performed using T cells derived from 7 different donors. Each experiment was conducted in triplicate (technical replicates). Statistical analysis: Tukey's multiple comparisons test; ****≤0.0001. *Abbreviations: aPBMNCs: Activated Peripheral Blood Mononuclear Cells*

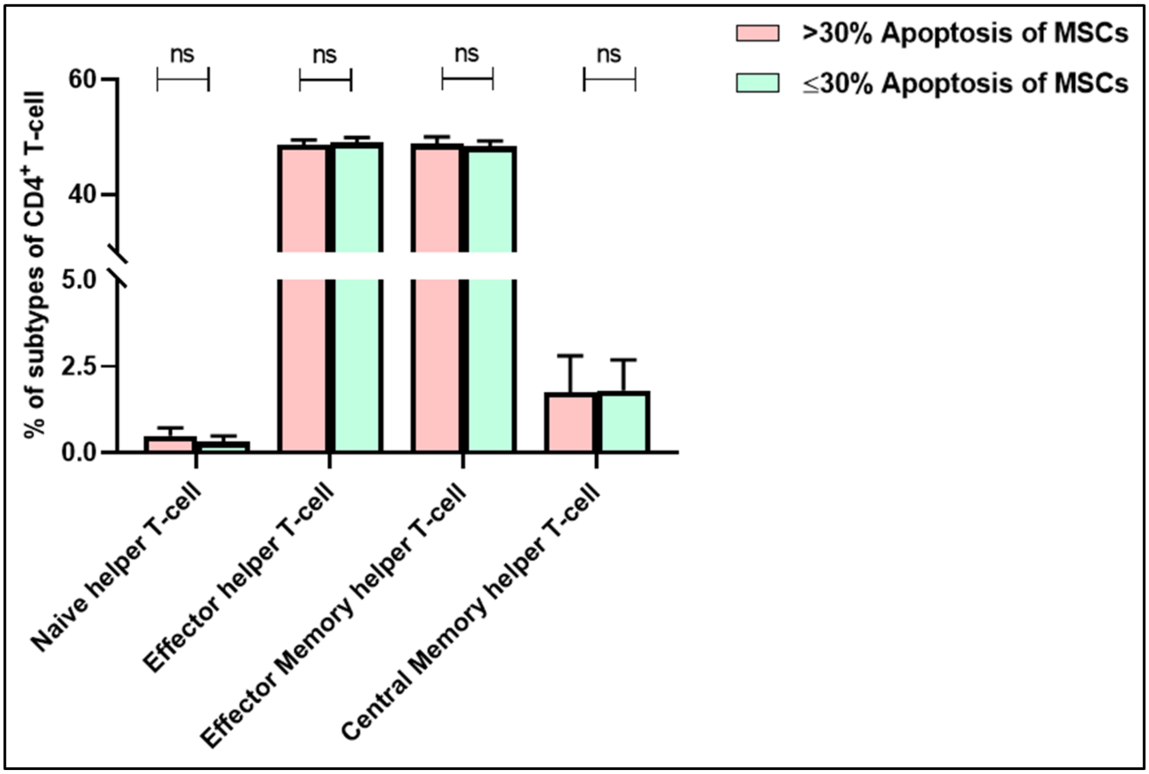

Figure S4: Distribution of different subtypes of CD4$^+$ T-cell in the co-culture of MSCs (BM-MSCs, BM-MSCs^{HYP}, WJ-MSCs, and WJ-MSCs$^{HYP)}$ and aGVHD patient-derived aPBMNCs. The bar graph represents the percentage of subtypes of CD4$^+$ T-cell. Data shown represent the Mean±S.D of 25 independent experiments performed with T-cell derived from 25 different donors (biological replicates), with each experiment conducted in triplicate (technical replicates). Statistical analysis: Tukey's multiple comparisons test. *Abbreviations: BM: Bone marrow; WJ: Wharton's Jelly; MSCs: Mesenchymal Stem Cells; HYP: Hypoxia-preconditioned; APO: Apoptosis; Eff: Efferocytosis; aPBMNCs: Activated Peripheral Blood Mononuclear Cells*

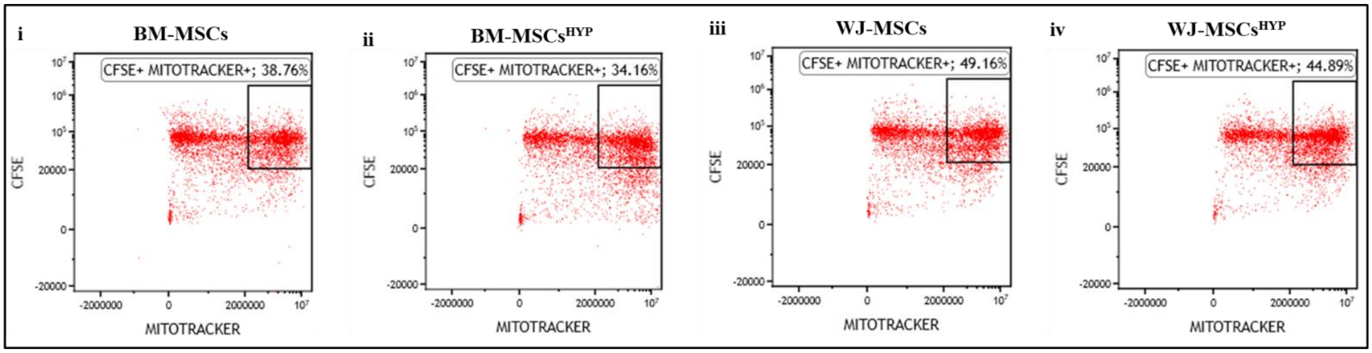

Figure S5: Representative dot plots showing mitochondrial transfer from MSCs to T-cell derived from aGVHD patients. The dot plots represent the percentage of double-positive ($CFSE^+Mitotracker^+$) populations in the co-culture of CFSE-labeled T-cell with Mitotracker-labeled i) BM-MSCs. ii) BM-$MSCs^{HYP}$. iii) WJ-MSCs. and (iv) WJ-$MSCs^{HYP}$.

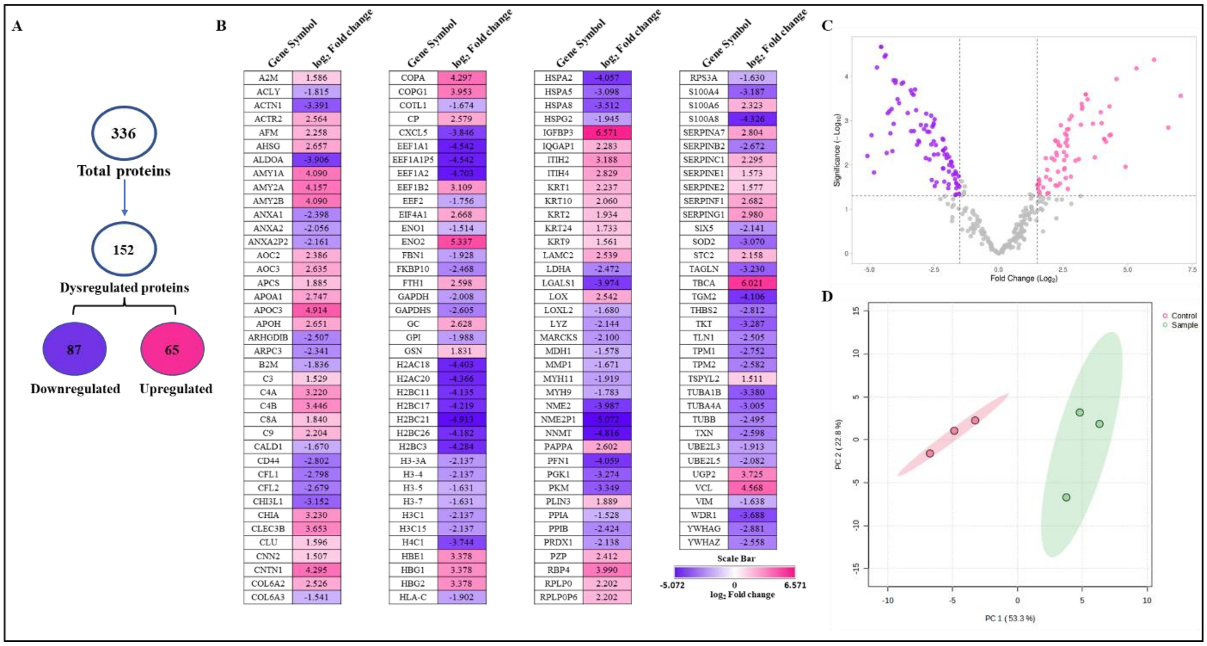

Figure S6: Label-free proteomics analysis of the WJ-MSCs and their direct co-culture with aPBMNCs using LC-MS/MS. A) A flow chart depicts the total number of identified and dysregulated proteins. B) A heat map shows the expression levels of dysregulated proteins. C) A volcano plot highlights differentially expressed proteins. D) Principal component analysis (PCA) demonstrates the good reproducibility of each biological replicate. Independent experiments were conducted with three different donors (biological replicates).

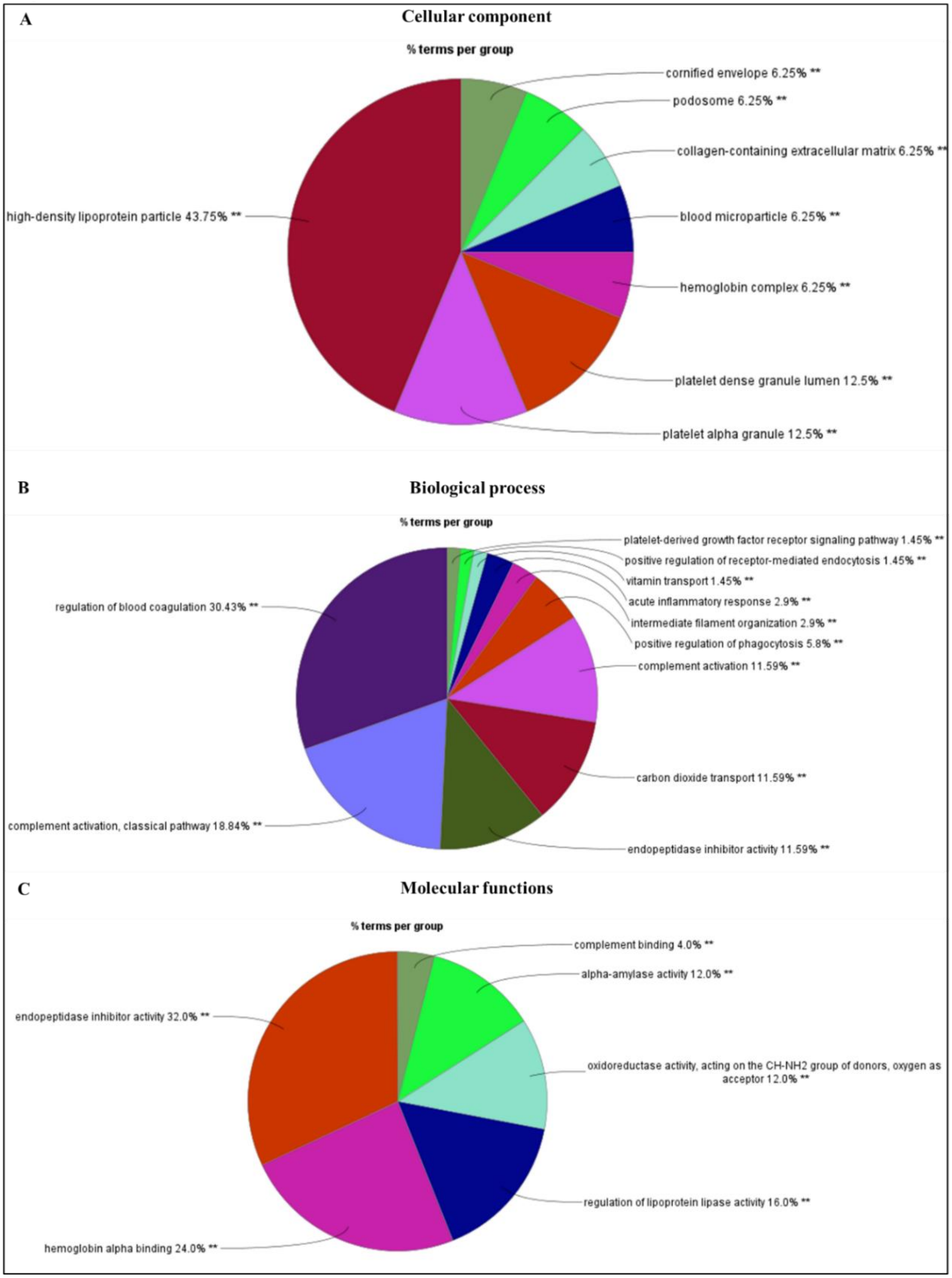

Figure S7: Gene Ontology (GO) analysis of upregulated proteins in WJ-MSCs compared to their co-culture with aPBMNCs. Pie charts depict A) Cellular component. B) Biological process. C) Molecular function. Data showed three independent experiments conducted with three different donors (biological replicates). **≤0.01

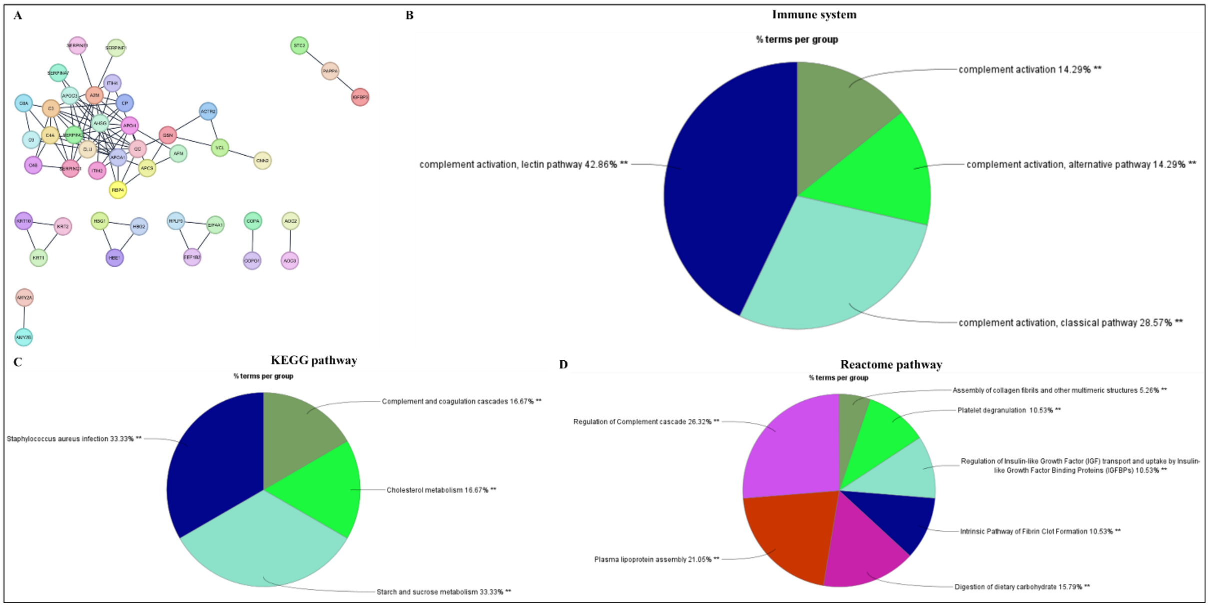

Figure S8: Functional enrichment analysis of upregulated proteins in WJ-MSCs compared to their co-culture with aPBMNCs. A) STRING network. Pie charts depict B) Immune system process. C) KEGG pathway. D) Reactome pathway. Data showed three independent experiments conducted with three different donors (biological replicates). **≤0.01

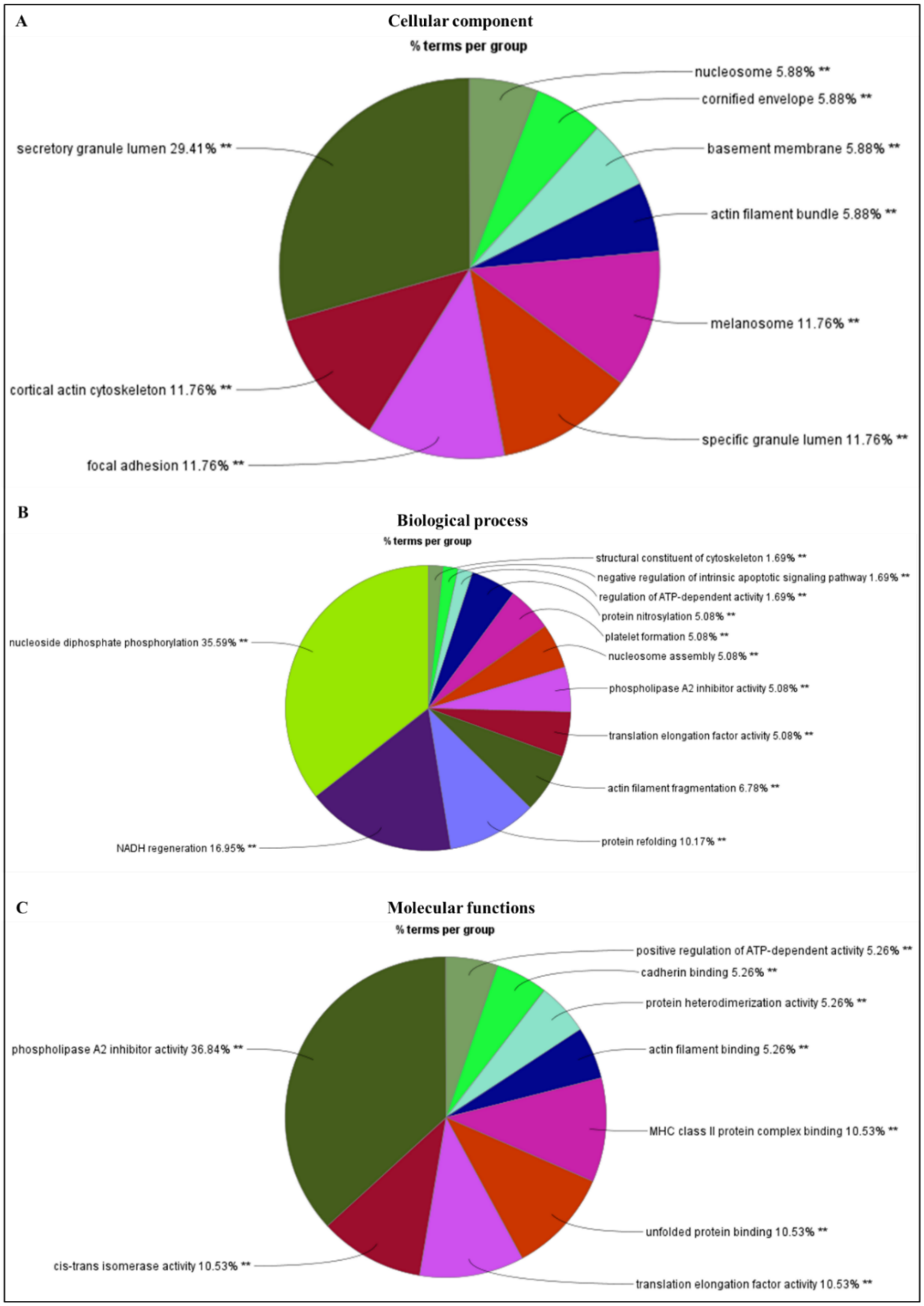

Figure S9: Gene Ontology (GO) analysis of downregulated proteins in WJ-MSCs compared to their co-culture with aPBMNCs. Pie charts depict A) Cellular component. B) Biological process. C) Molecular function. Data showed three independent experiments conducted with three different donors (biological replicates).

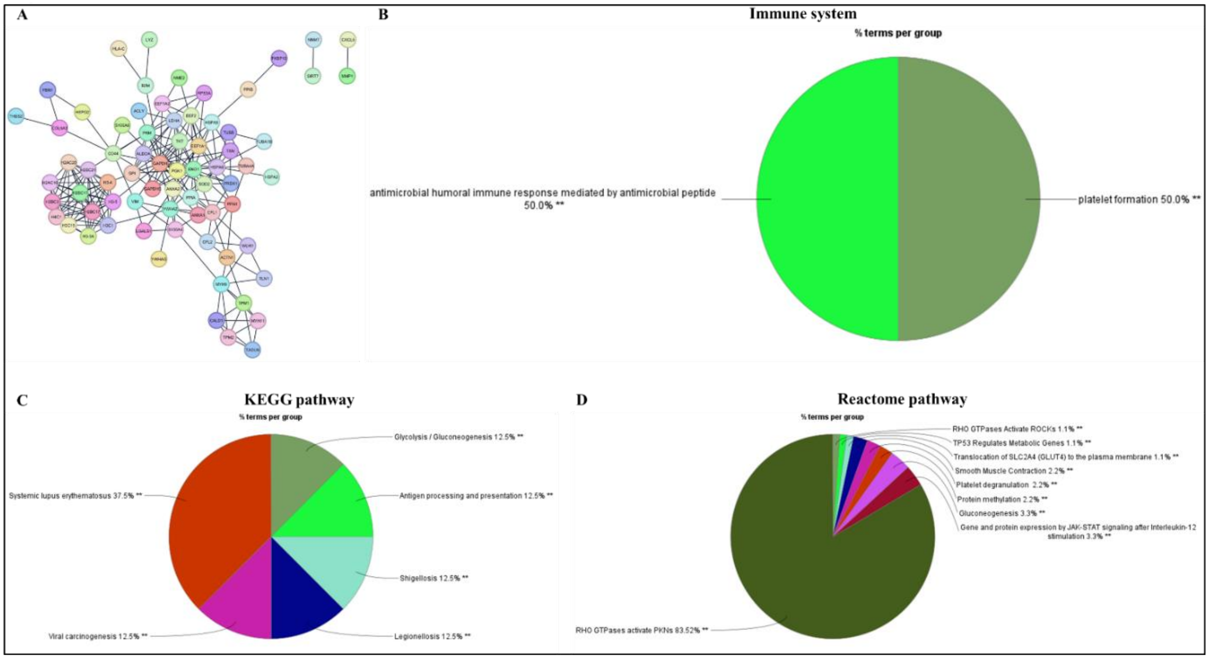

Figure S10: Functional enrichment analysis of downregulated proteins in WJ-MSCs compared to their co-culture with aPBMNCs. A) STRING network. Pie charts depict B) Immune system process. C) KEGG pathway. D) Reactome pathway. Data showed three independent experiments with three different donors (biological replicates).

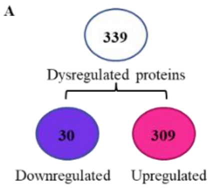

B

Gene Symbol	log2 Fold change	Gene Symbol	log2 Fold change	Gene Symbol	log2 Fold change	Gene Symbol	log2 Fold change	Gene Symbol	log2 Fold change	Gene Symbol	log2 Fold change	Gene Symbol	log2 Fold change	Gene Symbol	log2 Fold change
ACAA2	1.54	CAP1	7.266	EIF4G1	4.697	HNRNPA1	3.979	KRT9	1.611	PCBP1	2.57	PTX3	-2.778	TAGLN	3.3
ACLY	1.97	CAPG	5.455	EIF4H	1.655	HNRNPA2B1	3.005	KTN1	7.698	PCNA	2.595	RAB11B	1.987	TAGLN2	2.58
ACTA1	4.585	CAPN2	-1.726	EIF5A	3.566	HNRNPC	4.678	KYNU	1.9	PCOLCE	-2.887	RAD23B	2.924	TALDO1	5.409
ACTN1	3.222	CAPZB	3.915	EIF6	2.154	HNRNPF	3.764	LAMB1	2.5	PDCD5	3.001	RAN	3.004	TARS1	4.189
ACTN4	6.25	CCT4	2.745	EML2	-1.825	HNRNPH1	4.302	LCP1	5.196	PDCD6IP	2.265	RANGAP1	3.209	TCP1	2.124
ADA	2.43	CCT6A	1.726	ENO1	1.78	HNRNPK	4.799	LDHA	4.431	PDIA3	5.775	RBM39	2.423	THUMPD1	2.042
ADGRE5	1.844	CCT7	2.653	ENOPH1	1.864	HNRNPM	4.285	LDHB	4.529	PDIA6	5.716	RBMX	2.268	TKT	2.833
ADSS2	6.328	CD44	3.997	EPB41	3.437	HNRNPU	2.897	LGALS1	3.515	PEBP1	3.453	RDX	2.522	TLN1	5.303
AFP	1.98	CD63	3.493	ESYT1	1.8	HP	-2.742	LILRA3	-1.503	PFDN2	4.108	RHOA	3.521	TPD52L2	5.027
AHNAK	2.811	CDC42	2.184	EVL	3.39	HPRT1	3.841	LMNB1	3.741	PFN1	6.874	RPIA	5.304	TPI1	8.799
ALDOA	4.315	CFL1	2.856	FBLN1	1.768	HSP90B1	2.099	LMNB2	2.558	PGAM1	6.564	RPL14	3.773	TPM2	3.827
ALDOC	2.222	CHIA	1.716	FEN1	1.951	HSPA4	2.265	LRRFIP1	-2.731	PGM2	2.12	RPS21	2.135	TPM3	2.386
AMY1A	1.573	CLSTN1	2.913	FERMT3	2.17	HSPA5	3.625	LSM8	2.856	PGP	1.715	RPS3	2.702	TPM4	1.896
ANP32A	-1.542	CLTA	3.485	FKBP10	4.63	HSPA8	5.164	LSP1	3.786	PITPNA	-2.117	RPSA	6.024	TPR	2.083
ANP32B	2.011	CNDP2	1.718	FLG2	-1.766	HSPA9	2.999	MAPRE1	2.499	PKM	3.827	RRBP1	2.555	TUBA1A	3.287
ANPEP	3.38	CNN2	2.548	FLII	2.133	HSPD1	3.554	MARCKS	2.671	PLEC	2.125	RSU1	2.921	TUBA4A	1.866
ANXA1	4.089	CNTN1	1.521	FLNA	4.347	HSPE1	1.666	MCM2	2.369	PLIN3	4.392	S100A11	2.843	TUBB	3.924
ANXA2	1.91	COL12A1	-1.759	FSTL1	-3.078	HYOU1	2.56	MCM3	3.419	PNP	4.316	S100A8	2.493	TUBB4B	3.809
ANXA5	3.67	COL1A1	[illegible]	FUBP1	2.018	IDH1	-4.103	MDH1	3.45	PPBP	2.796	S100A9	4.256	TXN	3.514
ANXA6	5.706	COL1A2	-4.249	G3BP1	2.802	IGF2R	1.786	MDH2	6.481	PPIA	4.87	SAA4	4.257	TYMP	5.097
AP1B1	1.687	COL6A3	3.646	GAPDH	4.349	IGHM	3.447	METAP2	3.423	PPIB	5.141	SAE1	-3.056	UBA1	3.992
AP1M1	2.132	COPS4	1.72	GARS1	2.532	IGLL5	2.921	MGAT1	2.136	PPP1R18	2.496	SDF4	7.185	UBA52	3.992
APEH	3.154	COTL1	4.705	GBP1	1.567	IL16	2.845	MMP1	3.023	PPP2R1A	2.993	SERPINB1	3.162	UBE2K	3.466
APOB	1.719	CPA4	-4.172	GDI1	5.887	IL1B	-2.36	MMP2	-2.601	PRDX1	4.457	SERPINB2	5.348	VAPA	1.909
APOE	2.699	CRYZ	1.852	GDI2	2.74	IL6	4.206	MSN	1.756	PRDX2	3.663	SERPINB9	2.451	VCAN	-2.547
APOM	1.76	CSTB	6.866	GGH	1.827	ILF2	-1.732	MVP	2.583	PRDX5	1.613	SERPINC1	1.521	VCL	3.88
ARCN1	2.18	CTBS	-3.396	GLUL	2.709	ILF3	2.199	MYH9	3.676	PRDX6	1.654	SERPINE2	-2.497	VCP	2.13
ARF1	3.757	CTSB	2.283	GNS	3.454	IQGAP1	2.097	MYL12B	4.842	PRKCSH	2.998	SERPINF1	1.833	VIM	4.513
ARHGDIA	2.091	CTSD	3.581	GPI	4.32	ITGAL	1.522	MYL6	7.404	PRSS3	1.519	SERPING1	1.85	WARS1	1.714
ARHGDIB	1.996	DCD	2.339	GRB2	2.02	ITIH1	1.914	NAMPT	3.426	PSMA2	8.951	SERPINH1	2.188	WDR1	1.665
ARPC1B	2.252	DEF6	1.885	GSR	2.824	ITIH4	2.278	NAPA	3.511	PSMA3	4.606	SFPQ	5.587	XPO1	1.958
B2M	3.942	DNAJC8	1.72	GSS	3.872	JCHAIN	2.753	NARS1	4.743	PSMA7	3.053	SH3BGRL	3.021	XRCC5	2.854
BASP1	1.632	DNPEP	-1.535	GSTO1	2.971	JUP	2.447	NCL	2.486	PSMB1	1.771	SNRPD2	-1.735	YBX1	2.462
BCAP31	1.968	DSP	1.532	H2AFY	3.033	KPNB1	3.985	NME2	6.436	PSMB2	1.825	SNRPE	1.666	YWHAB	7.944
BLVRB	2.41	EDF1	3.655	H4C1	2.709	KPRP	1.702	NPC2	1.756	PSMB3	4.019	SOD1	1.653	YWHAE	6.282
BSG	2.008	EEA1	2.211	HBA1	3.346	KRT10	1.546	NRP2	1.503	PSMB4	5.298	SPARC	-2.636	YWHAQ	2.973
C11orf54	1.822	EEF1A1	4.819	HBB	4.996	KRT14	2.683	NUMA1	1.846	PSMB8	3.942	SPOCK1	-2.024	YWHAZ	5.964
C2	2.633	EEF2	2.285	HCLS1	1.622	KRT17	2.968	NUTF2	3.164	PSMD2	2.23	SPOCK2	2.204	ZYX	1.666
CA1	4.849	EHD4	3.104	HEXA	2.379	KRT2	1.792	P4HB	1.944	PSME1	4.32	SPTAN1	2.851		
CALM3	2.4	EIF3F	6.218	HLA-B	3.602	KRT6A	2.083	PA2G4	1.509	PSME2	3.99	SPTBN1	3.884		
CALR	2.391	EIF3H	2.257	HLA-C	6.318	KRT6B	2.573	PABPC1	3.771	PTBP1	7.848	STAT1	3.595		
CALU	1.831	EIF3L	-3.164	HLA-DRB1	-3.154	KRT77	1.582	PARP1	1.676	PTMA	2.54	STIP1	3.249		
CAND1	1.826	EIF4A2	2.054	HMGB1	2.088	KRT78	1.966	PAXX	-1.72	PTPRC	5.066	STMN1	4.567		

Scale Bar

-4.883 0 8.951

log_2 Fold change

Figure S11: Label-free proteomics analysis of BM-MSCs and their co-culture with aPBMNCs using LC-MS/MS. A) A flow chart depicts the total number of identified and dysregulated proteins. B) A heat map shows the expression levels of dysregulated proteins. An experiment was conducted with a single donor.

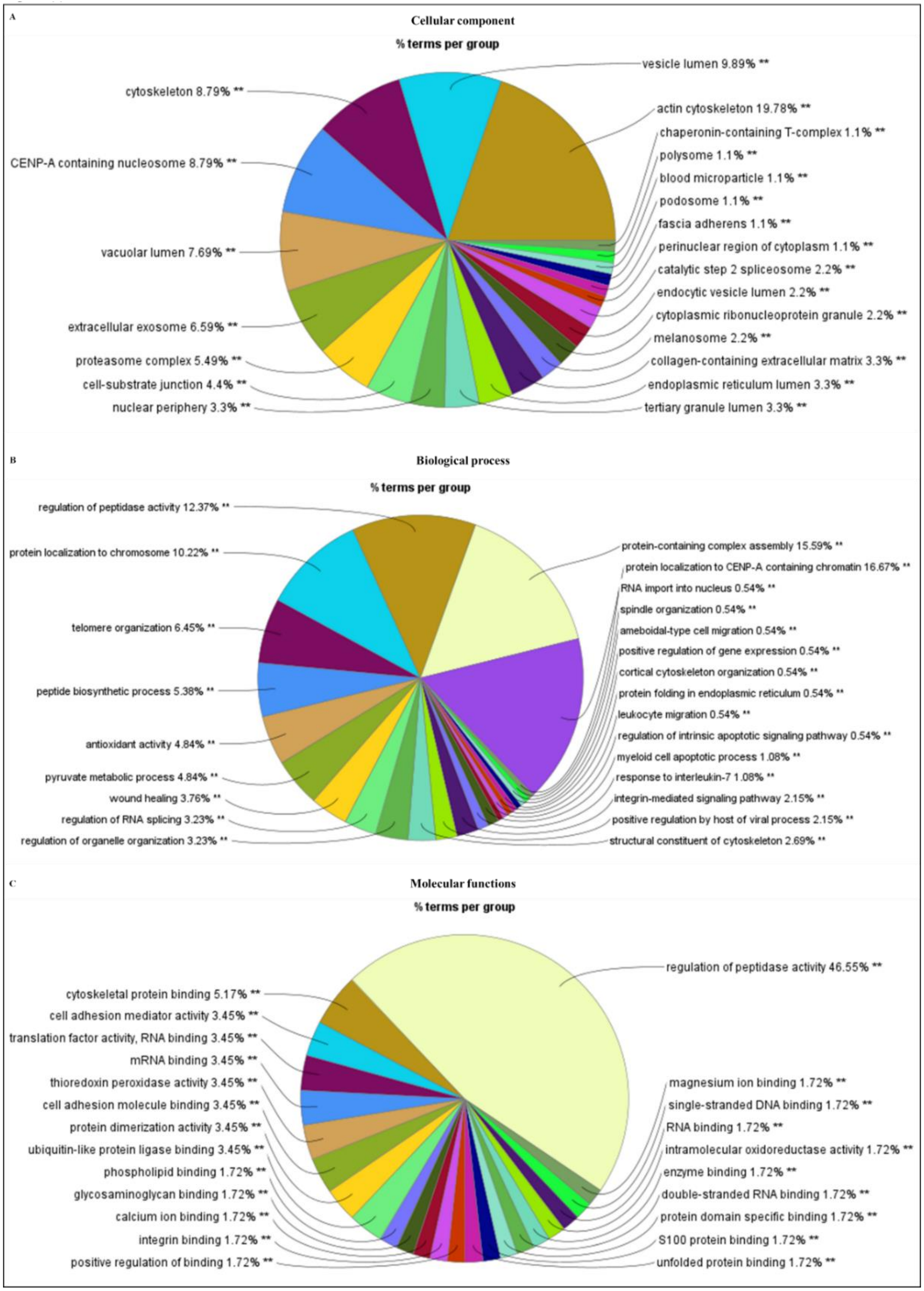

Figure S12: Gene Ontology (GO) analysis of upregulated proteins in the co-culture of BM-MSCs and aPBMNCs. Pie charts depict A) Cellular component. B) Biological process. C) Molecular function. Data showed a single independent experiment conducted with a single donor. **≤0.01

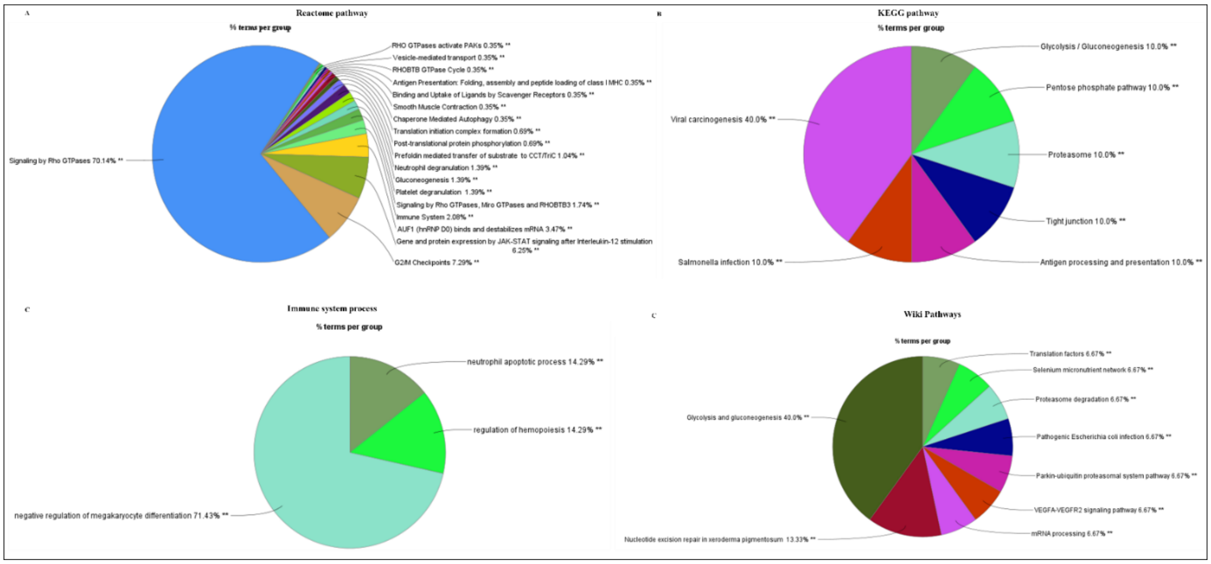

Figure S13: Functional enrichment analysis of upregulated proteins in the co-culture of BM-MSCs and aPBMNCs. Pie charts depict A) Reactome pathway. B) KEGG pathway. C) Immune system process. D) WiKi pathway. Data showed a single independent experiment conducted with a single donor. **≤0.01

Supplemental tables

Table S1: Comparative immunomodulatory potential of BM-MSCs and WJ-MSCs (naïve, hypoxia-preconditioned). Data shown represent the Mean±S.D of 25 independent experiments from 25 different donors (biological replicates), with each experiment conducted in triplicate (technical replicates). Statistical analysis: Tukey's multiple comparisons test; *≤0.05; **≤0.01; ***≤0.001; ****≤0.0001.

	Control	BM-MSCs		WJ-MSCs		p-value	p-value	p-value	p-value	p-value
		Naive	Hypoxic	Naive	Hypoxic	Control vs BM	BM vs WJ	BM vs BM^{HYP}	WJ vs WJ^{HYP}	BM^{HYP} vs WJ^{HYP}
$CD3^+$ T-cell proliferation	95.067%	88.852%	84.134%	76.851%	64.957%	0.0047	≤0.0001	0.1153	≤0.0001	≤0.0001
$CD4^+/CD8^+$ T-cell ratio	0.302	0.318	0.357	0.389	0.545	0.9138	0.0034	0.2387	≤0.0001	≤0.0001
γδ/αβ T-cell ratio	0.004	0.005	0.007	0.006	0.009	0.0291	0.0893	0.0003	0.0003	0.0982
Tregs	2.692%	3.150%	5.856%	4.21%	10.08%	0.5756	0.0184	≤0.0001	≤0.0001	≤0.0001
Th1/Th2 ratio	24.418	22.424	19.52	20.8788	16.2474	0.4098	0.6455	0.1089	0.0041	0.0571
Th1/Th17 ratio	0.516	0.748	1.178	1.000	2.252	0.7064	0.6410	0.1644	≤0.0001	≤0.0001
Th1/Th9 ratio	37.252	30.338	19.42	25.526	11.598	≤0.0001	0.0011	≤0.0001	≤0.0001	≤0.0001
M1	93.7045%	83.564%	78.027%	66.958%	57.639%	0.0027	≤0.0001	0.2502	0.0073	≤0.0001
Arginase-1^+ M2	1.8371	6.9895	13.6615	19.1475	30.491	0.0541	≤0.0001	0.0051	≤0.0001	≤0.0001

Table S2: Demographic and clinical characteristics of patients with acute graft-versus-host disease included in the study cohort. Age is presented as median years. Patient distribution according to sex, underlying diagnosis, donor type, conditioning regimen, total body irradiation status, GVHD grade, and GVHD prophylaxis regimen is shown. *Abbreviations: AML: acute myeloid leukemia; ALL: acute lymphoblastic leukemia; CLL: chronic lymphocytic leukemia; MDS: myelodysplastic syndrome; ATG: anti-thymocyte globulin; PTCy: post-transplant cyclophosphamide; GVHD: graft-versus-host disease; CNI: calcineurin inhibitor; MTX: methotrexate; MMF: mycophenolate mofetil.*

**Age in years, median**	
Patient	22
**Sex**	
Male	20
Female	05
**Diagnosis**	
AML	09
ALL	03
CLL	01
Aplastic Anemia	06
MDS	02
Others	04
**Donor**	
Haploidentical	07
Matched sibling donor	18
**Conditioning regimen**	
ATG only	04
ATG+PTCy	01
PTCy only	04
Others	16
**Total body irradiation**	
Yes	01
No	24
**GVHD (grade II-IV)**	
**Grade II**	09
**Grade III**	05
**Grade IV**	10
**Grades III and IV both**	01
**GVHD prophylaxis**	
CNI+MTX+/-Steroids	16
CNI+MMF+/-Steroids	09
